# Ab Initio Design of Molecular Qubits with Electric Field Control

**DOI:** 10.1021/jacs.4c09109

**Published:** 2024-09-05

**Authors:** William T. Morrillo, Herbert I. J. Cumming, Andrea Mattioni, Jakob K. Staab, Nicholas F. Chilton

**Affiliations:** † Department of Chemistry, The University of Manchester, Oxford Road, Manchester M13 9PL, U.K.; ‡ Research School of Chemistry, The Australian National University, Canberra 2601, Australia

## Abstract

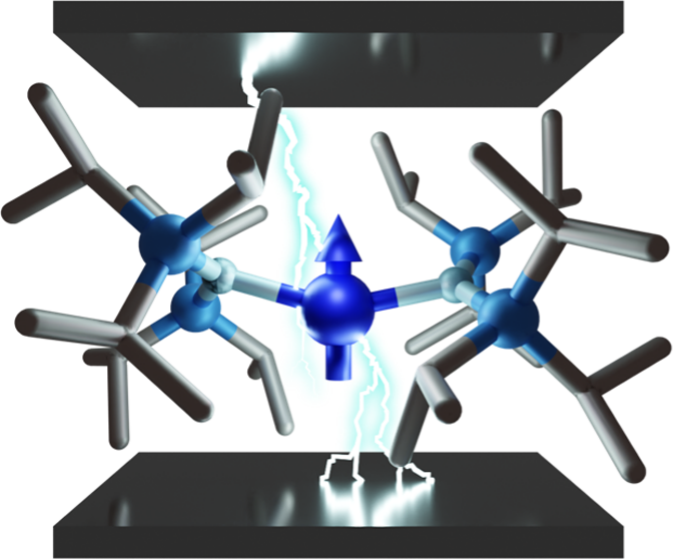

Current scalable quantum computers require large footprints and complex interconnections due to the design of superconducting qubits. While this architecture is competitive, molecular qubits offer a promising alternative due to their atomic scale and tuneable properties through chemical design. The use of electric fields to precisely, selectively and coherently manipulate molecular spins with resonant pulses has the potential to solve the experimental limitations of current molecular spin manipulation techniques such as electron paramagnetic resonance (EPR) spectroscopy. EPR can only address a macroscopic ensemble of molecules, defeating the inherent benefits of molecule-based quantum information. Hence, numerous experiments have been performed using EPR in combination with electric fields to demonstrate coherent spin manipulation. In this work, we explore the underlying theory of spin-electric coupling in lanthanide molecules, and outline ab initio methods to design molecules with enhanced electric field responses. We show how structural distortions arising from electric fields generate coupling elements in the crystal field Hamiltonian within a Kramers doublet ground state and demonstrate the impact of molecular geometry on this phenomenon. We use perturbation theory to rationalize the magnetic and electric field orientation dependence of the spin-electric coupling. We use pseudo-symmetry point groups to decompose molecular distortions to understand the role that symmetry has on spin-electric coupling. Finally, we present an analytical electric field model of structural perturbations that provides large savings in computational expense and allows for the investigation of experimentally accessible electric field magnitudes which cannot be accessed using common ab initio methods.

## 1 Introduction

Molecular spin qubits show promise in quantum information science.^1−11^ A key benefit is that their quantum properties can be tuned through chemistry such that different operation frequencies or gates can be realized.^3,12^ Furthermore, molecular spin systems can be designed with more than two spin degrees of freedom and thus can be used as qudits with arbitrary Hilbert space dimension *d*. This is exemplified by hyperfine-coupled electron and nuclear spin states, which have been proposed as a basis for fault tolerant qubit manipulation,^13,14^ used to implement multiqubit algorithms and as quantum simulators.^15−19^

For a qubit to be considered a suitable candidate for quantum information processing it must meet the DiVincenzo criteria;^20^ in particular, it needs to be individually addressable and measurable. Electron paramagnetic resonance (EPR) spectroscopy is currently the most commonly utilized technique for manipulating molecular qubits. However, pulsed EPR experiments with GHz microwave frequencies have wavelengths on the order of centimeters, and therefore do not provide the necessary spatial selectivity to address a single qubit. While this is a limitation of spatial addressing, in principle individual qubits could be spectrally addressed if their frequencies were distinct enough, which has been proposed and demonstrated before.^21^ Here, however, the limitation is that transitions need to be sharp enough, and at distinct enough frequencies that fit within the microwave amplifier and resonator bandwidths, which is usually only several hundred MHz. Furthermore, scaling to larger arrays of qubits leads to spectral crowding which may impose rather low limits on the number of individually addressable qubits. Electric field manipulation could provide far greater spatial control, provided the fundamental spin-electric field control mechanisms can be understood and built into molecular qubits.

To that end, recent literature has demonstrated effective manipulation of molecular spin qubits using applied electric fields. Clock transitions in [Ho(W_5_O_18_)_2_]^9–^, arising due to molecular distortions breaking local pseudo-D_4d_ symmetry,^22^ have been recently shown to have a linear spin-electric field coupling.^23^ Other work has shown magneto-electric coupling in [Fe_3_O(PhCOO)_6_(py)_3_]ClO_4_·py in which electric field pulses were incorporated into an EPR pulse sequence that induced a spin flip attributed to coupling of the electric field due to the spin chirality of the Fe_3_ triangle through spin–orbit interactions.^24^

Pertaining to employing the high-spatial resolution afforded by electric field techniques, scanning tunneling microscopy (STM) is widely used for mapping and imaging surfaces, and has more recently been used to probe submolecular properties. In the present context, inelastic tunneling spectroscopy has been used to measure the spin polarization of Co, Fe, and Ho atoms on a surface as well as using a Ti sensor neighboring a Fe atom containing a half integer spin to read the single atom spins without direct measurement of the Fe atom itself.^25−28^ The STM technique has been further applied to a pair of iron phthalocyanine complexes on a bilayer of MgO on a Ag(100) surface, where single molecule EPR was driven by the manipulation of the spin of [FePc]^−^, localized on the iron, using an STM tip.^26^ Furthermore, this technique was used to probe the role of the phthalocyanine ligands and their coordination geometry on the [FePc]^−^–[FePc]^−^ exchange coupling. In other work, spin-polarized STM was used to drive EPR-like transitions to investigate the *T*_2_ times of Fe atoms on a MgO/Ag(001) surface. It was determined that the signal has a linear dependence on the tunneling current, and it was proposed that this technique could be used to investigate, control, and distinguish decoherence on a single atom basis for quantum sensing applications.^27^

STM has also been shown to be able to read and write the electronic states of an individual Ho atom on a MgO/Ag(100) surface using tunneling magnetoresistance and pulsed current STM,^29^ as well as to measure the largest zero-field splitting of a Co atom at 58 meV on a MgO(100) surface.^28^ Thus, there is ample precedent for spatial control of electric fields and associated spectroscopy on the submolecular scale, and thus manipulation of individual molecular spin qubits using highly localized electric fields is a possibility, if the coupling can be built in via molecular design. While existing experimental work has shown that electric fields can manipulate molecular spin qubits, the mechanism of how the spin-electric coupling arises and how this coupling can be engineered is not well understood.^23,24,30,31^

Furthermore, it has been shown that external electric fields can modulate the magnetization of a ferroelectric single-molecule magnet through the opening of a tunneling gap enhancing the magnetic relaxation via quantum tunneling of the magnetization (QTM).^32^ This can be seen by the decrease in magnetization at zero magnetic field upon the application of an electric field. Electric fields have also been shown to affect the long-range ordering of multiferroic materials,^33−35^ where the magnetic ordering is influenced by applied electric fields in the form of a spin-wave. In that case, the magneto-electric coupling results in a shift of the spin-wave frequency as a function of electric field strength.

The dependence on the magneto-electric coupling as a function of the magnitude of the applied electric field has also been the subject of computational studies. In the case of LiFePO_4_, a linear magneto-electric coupling was found to arise from spin–orbit interactions, lifting the quenching of the 3d orbitals of Fe^2+^, and allowing structural distortions introduced by an applied electric field to influence the orbital angular momentum.^36^ As we will discuss in greater detail herein, the breaking of symmetries plays a crucial role in enabling spin-electric coupling. On the one hand, electronic states in Kramers ions are doubly degenerate owing to time-reversal symmetry of the molecular Hamiltonian. As electric fields preserve time-reversal symmetry, geometric distortions are unable to generate couplings between Kramers conjugate states, unless the time-reversal symmetry is broken by a magnetic field. On the other hand, electric fields break spatial inversion symmetry, distorting the molecular geometry and inducing an electric dipole moment. We shall see that the electric dipole generated by structural distortions plays a fundamental role in determining the spin-electric coupling. Beyond these fundamental principles, there are as yet no design guidelines for synthetic chemists to prepare molecules that are predisposed toward electric-field spin control.

In this work we use ab initio computational methods and perturbation theory to explore how spin-electric field coupling may arise from structural perturbations in [Tm(N(Si^i^Pr_3_)_2_)_2_] (herein Tm(N^††^)_2_), which has a pseudo-spin *S̃* = 1/2 ground state (*and also a I* = 1/2 nuclear spin, but we do not discuss hyperfine effects herein).^37^ We present an analytical model to compute distortions under an electric field from a single shot ab initio computation, which vastly reduces computational cost and allows access to experimentally relevant electric field strengths, not currently accessible with common ab initio simulations of applied electric fields. We then investigate how molecular design influences the strength of spin-electric coupling, studying a series of “toy” molecules characterized by different coordination geometries. This allows us to identify the relationship between the molecular dipole moment, geometry, (pseudo-)symmetry and the spin-electric coupling. The prospect of these design criteria is the ability to design molecular qubits such that the coupling between the *S̃* = 1/2 basis states is large enough to facilitate an electric-field driven spin flip with a resonant oscillating electric field π-pulse in a small static magnetic field.

## 2 Experimental Section

The molecular structure of [Tm(N(Si^i^Pr_3_)_2_)_2_] was isolated from the crystal structure obtained from the CCDC.^37^ The structure was then optimized in the gas phase using density-functional theory (DFT) in Gaussian 16 revision C01 with the PBE functional and the GD3 dispersion correction.^38−40^ The Tm atom was treated using the 4f-in-core Stuttgart RSC effective core potential (ECP).^41−44^ All ligand atoms were treated using double-ζ cc-pVDZ basis sets.^45^ Electric field optimization calculations were performed in Gaussian using the “Field” keyword starting from the gas-phase optimized equilibrium geometry. Calculations were performed for a range of field strengths spanning 3 × 10^8^–3 × 10^9^ V m^–1^ for each Cartesian direction *x*, *y*, *z*. This range is dictated on the lower bound by limits in Gaussian 16, and the upper bound being an order of magnitude larger. It should be noted that this range of field strengths are attainable experimentally with STM, but not explored in existing research on molecular spin-electric coupling where electric field strengths are of the order of 10^6^ V m^–1^.^24^ “Toy” molecules were designed in Avogadro and initially optimized using the UFF force field.^46,47^ Structures were then optimized in Gaussian using the Stuttgart RSC ECP basis set on the Dy atom and a double-ζ cc-pVDZ basis set for all other atoms.

Complete active space self-consistent field spin–orbit (CASSCF-SO) calculations were performed in OpenMolcas v22.06 using a minimal 13 electrons in 7 orbital active space, modeling the 13 4f electrons of Tm^2+^ in the 7 4f orbitals.^48−50^ For all toy model structures, the active space consisted of the 9 4f electrons of Dy^3+^ in seven 4f orbitals. Electronic states were computed at the CASSCF level of theory including scalar relativistic effects using the second-order Douglas–Kroll Hamiltonian, and spin–orbit coupling using the AMFI approximation.^51,52^ The lanthanide metal centers were equipped with ANO-RCC-VTZP basis sets and all other atoms were equipped with ANO-RCC-VDZP basis sets. The resolution of identity approximation was employed and the acCD auxiliary basis was used to treat the two electron integrals.^53^ The crystal field parameters were obtained by projection of the ab initio Hamiltonian onto a crystal field model Hamiltonian using angmom_suite.^54^ We apply a weak magnetic field (320 mT) to the crystal field Hamiltonian as a Zeeman term to lift the degeneracy of doublet Kramers states and break the time reversal symmetry to allow for intra-Kramers doublet mixing in the electric field Hamiltonian.

To compute the crystal field Hamiltonians for the set of displaced geometries generated by the decomposition of ab initio calculated electric field distorted geometries into the new symmetry adapted coordinate bases (see Supporting Information), we use the linear vibronic coupling (LVC) methodology in spin_phonon_suite.^55,56^ To do so, first the gas phase equilibrium geometry and vibrational modes of Tm(N^††^)_2_ were computed in Gaussian 16. A CAS(13,7)SCF calculation was performed at the equilibrium geometry, followed by gradient and nonadiabatic coupling calculations using OpenMolcas. The equilibrium CASSCF electronic states, molecular gradients and nonadiabatic couplings were used to parametrize the LVC model and subsequently compute the crystal field parameters (*B*_*k*_^*q*^) for each 3*N* symmetry adapted coordinate displacement, which allows for the evaluation of contributions each symmetry irreducible representation of the point group has to the total spin-electric coupling.

The analytic electric field model is implemented into spin_phonon_suite and is performed based on a harmonic approximation of the potential energy surface. Our gas phase implementation interfacing with Gaussian 16 uses a frequency calculation that computes the derivative of the electric dipole moment with respect to atomic displacements, ∇r⃗μ⃗ which is returned as a [3*N* × 3] matrix. The electric dipole derivatives and the Hessian, ***H***, are used to calculate analytically the atomic displacements due to an applied electric field using the model described in S1.

We define the electric field perturbation Hamiltonian ***H***_*E⃗*_ in the following manner;

(i) the crystal field Hamiltonian ***H***_CF_ at equilibrium geometry is constructed as a linear combination of Stevens operators ***O***_*k*_^*q*^ of rank *k* and order *q*, weighted by crystal field parameters *B*_*k*_^*q*^ for even *k* = 2, 4, 6 terms of the ^2^F_7/2_ manifold
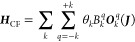
1
(ii) The Zeeman Hamiltonian is constructed from the total angular momentum operator of the ^2^F_7/2_ multiplet as
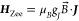
2
(iii) The equilibrium Hamiltonian is then formed from the summation of the crystal field and Zeeman Hamiltonians and is diagonalized as
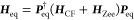
3where ***P***_eq_ is the unitary matrix that transforms the Hamiltonian from the total angular momentum basis into its eigenstate basis. The crystal field Hamiltonian of a structure distorted by an applied electric field (***H***_CF_^dist^) is defined in the total angular momentum basis using the same methodology as for ***H***_CF_. The Zeeman term is then added and the resulting Hamiltonian is transformed into the equilibrium eigenstate basis as
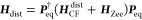
4The effect of the electric field in the eigenstate basis of ***H***_eq_ is then be described by the Hamiltonian
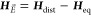
5

We note that the transformation ***P***_eq_ breaks time-reversal symmetry due to the presence of the Zeeman term; this is essential for allowing the time-reversal symmetric electric field to couple states within the same doublet, as we will discuss later in more detail. As a result, ***H_E⃗_*** presents both diagonal and off-diagonal elements, describing respectively energy shifts and couplings induced by the electric field *E⃗*.

All programs used (spin_phonon_suite, molcas_suite and angmom_suite) are available open source via the Chilton Group Gitlab page or for download via the python package manager on pypi.^54,56,57^

## 3 Results and Discussion

### 3.1 Electric Field Manipulation of [Tm(N(Si^i^Pr_3_)_2_)_2_]

**Figure 1:**
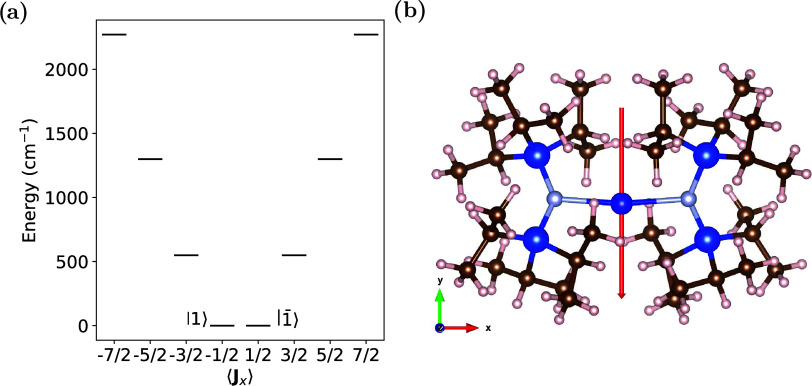
(a) Ab initio calculated spin–orbit states of Tm(N^††^)_2_ and their projection of the total angular momentum on ***J***_*x*_. The large energy separation between the ground first excited Kramers doublet allows for this system to be considered as a *S̃* = 1/2 spin qubit with the basis states consisting of the ground Kramers doublet (|1⟩, |1̅⟩). (b) Gas phase optimized structure of Tm(N^††^)_2_ containing two bis,tris-isopropylsilylamide ligands coordinated to a Tm^2+^ ion (Tm: dark blue; N: silver; Si: blue; C: brown; H: white). The molecular electric dipole vector is shown by the red arrow. Structure visualization was performed using VESTA.^58^

Tm(N^††^)_2_ was chosen as an initial candidate for investigating the potential for spin-electric coupling and coherent electric field control due to its pseudo-spin *S̃* = 1/2 Kramers doublet ground state, its neutral electric charge, and its lack of inversion symmetry due to a nonlinear N–Tm–N bond angle,^37^ which crucially gives rise to a permanent electric dipole. Moreover, naturally occurring Tm is isotopically pure (^169^Tm) with nuclear spin *I* = 1/2. This gives Tm(N^††^)_2_ a four-level hyperfine-split ground manifold which could be used to implement more complex quantum algorithms or for quantum error correction.^15,17^ However, we have chosen to study only the electronic degrees of freedom herein. Tm(N^††^)_2_ contains the Tm^2+^ ion, which shares a 4f^13^ electronic configuration with Yb^3+^. Given the buried nature of the 4f orbitals that results in near-degeneracy regardless of the molecular environment, this electronic configuration imbues the 4f electrons with unquenched orbital angular momentum, that strongly couples to the spin via spin–orbit coupling. This results in larger spin-electric coupling compared to systems containing d-block metals where the orbital angular momentum is usually quenched. The molecule has a pseudo-linear geometry with a *C*_2_ axis running perpendicular to the main molecular axis (N–Tm–N) which has an optimized bond angle of 171.98° (cf. 168.63° in the crystal structure). We define the Cartesian *x*, *y*, *z* coordinate system used here-on-in relative to the orientation of the Tm(N^††^)_2_ molecule shown in Figure 1b. The main molecular axis is oriented along *x* while the N–Tm–N angle defines the *xy*-plane, meaning that the permanent molecular electric dipole vector is oriented along *y*. The electric dipole arises from the positively charged Tm atom and the negatively charged ligand atoms. These factors suggest the molecule will have a predictable and quantifiable structural dependence on an electric field due to its geometry. Crucially, the electric dipole is perpendicular to the main magnetic anisotropy, so should have a substantial influence on the mixing of the zeroth-order electronic states. Tm^2+^ has a ^2^F_7/2_ ground term, resulting in four Kramers doublets split by crystal field effects. The energy separation between the ground and first excited doublet was calculated to be 550 cm^–1^ from a CASSCF-SO calculation using the optimized geometry, yielding an effective *S̃* = 1/2 system with basis states |1⟩, |1̅⟩ (Figure 1a).

**Figure 2:**
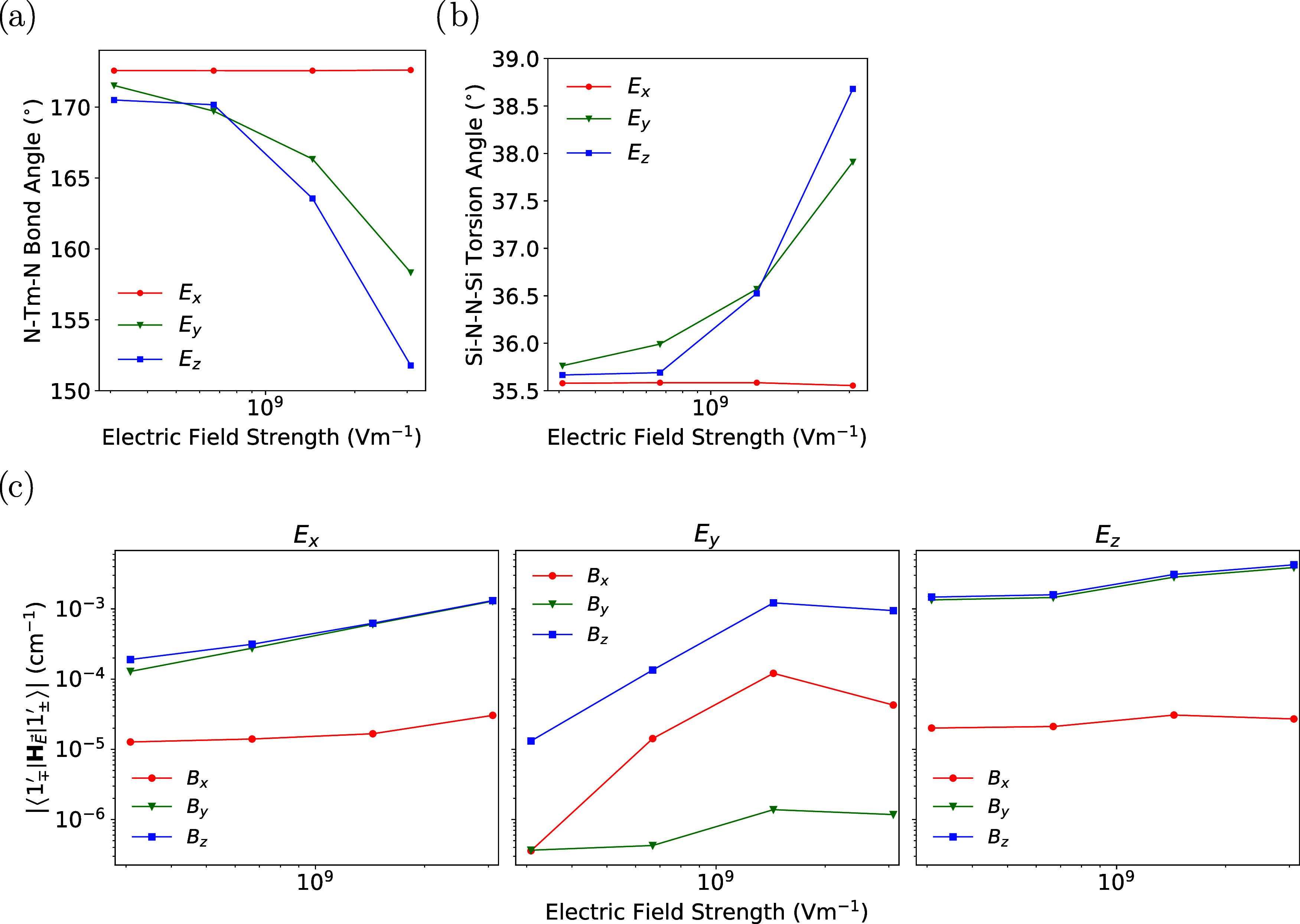
(a) N–Tm–N bond angle and (b) Si–N–N–Si torsion angle for geometry optimizations in the presence of varying strength and orientation of an applied external electric field. (c) The orientation dependence of the coupling element between the ground double states split by an applied magnetic field in each Cartesian direction for each electric field orientation.

Subsequent geometry optimization performed in the presence of an external electric field for field strengths evenly spread over an order of magnitude between 3 × 10^8^ and 3 × 10^9^ V m^–1^ for electric fields applied along each Cartesian direction, lead to clear structural distortions (Figure 2a,b). The N–Tm–N bond angle decreases from 171.98° at equilibrium to 158.3 and 151.8° for an electric field strength of 3 × 10^9^ V m^–1^ for the electric field oriented perpendicular to the main molecular axis, along *y* and *z* respectively. When the electric field is applied along the main molecular axis (*x*), where the electric dipole moment is approximately zero, the N–Tm–N bond angle only increases by 0.6° compared to the equilibrium geometry. Likewise, the Si–N–N–Si torsion angle, which measures the rotation of the amide ligands with respect to one another, follows similar trends; for electric field orientations perpendicular to the main molecular axis (*y*, *z*) the torsion angle increases from 35.44° at equilibrium to 37.9 and 38.7° for an electric field strength of 3 × 10^9^ V m^–1^ along *y* and *z* respectively, while there is a more minor change of 1.14° when the electric field is applied along *x*. The increase in Si–N–N–Si torsion angle can be rationalized as minimizing the steric clashing of the bulky isopropyl groups on the two ligands when the N–Tm–N bond angle is reduced. As expected, applying an electric field along the main molecular axis only results in slight distortions of the molecular geometry, which can be attributed to higher order multipolar effects.

As Tm(N^††^)_2_ has a Kramers electronic spin system, an electric-field-induced structural change that alters the crystal field potential cannot cause coupling within Kramers conjugate states, as the crystal-field Hamiltonian is even under time reversal symmetry. Hence, in this work we apply a nonzero static magnetic field to break the Kramers time reversal symmetry and enable the electric field Hamiltonian to have nonzero matrix elements within the ground electronic states; such a bias field is entirely experimentally viable and can be chosen to set the desired qubit operating frequency. Our definition of the electric field distortion Hamiltonian (***H***_CF_^dist^ – ***H***_CF_) and the overall spin-electric coupling Hamiltonian in the presence of the magnetic field (***H***_*E⃗*_) are defined in the Methods section (Section 2). In the following, we focus on the spin-electric coupling between the two lowest-energy eigenstates of ***H***_eq_, forming the Zeeman-split ground doublet |1_+_^′^⟩ and |1_–_^′^⟩.

The spin-electric coupling strength |⟨1_±_^′^|***H***_*E⃗*_|1_∓_^′^⟩| shows a strong dependence on the orientation and magnitude of the applied electric and magnetic fields *E⃗* and *B⃗* (Figure 2c). We note that the trends in the spin-electric coupling do not necessarily reflect the ones seen in the N–Tm–N bond angle and Si–N–N–Si torsion angle (Figure 2a,b), showing that the spin-electric coupling does not trivially depend on the magnitude of structural distortions alone. When the electric field is oriented along the main molecular axis, *x*, where the magnitude of the distortion with respect to the applied electric field strength is the smallest, we observe spin-electric couplings larger than those observed when the electric field is oriented parallel to the molecular dipole moment, *y*, which has significantly larger structural distortions (Figure 2c). However, when the electric field is oriented along *z*, also causing large structural distortions, we see the largest spin electric coupling. The smaller spin-electric couplings for an electric field along *y*, which is the pseudo-high-symmetry *C*_2_ axis, hints at the importance of the symmetry-breaking character of some of the structural deformations induced by *E⃗*. We will return to these subtleties in the next section. We also observe a large magnetic field orientation dependence of the spin-electric coupling. When the electric field is oriented along *x* or *z*, there is a distinct magnetic field orientation dependence with *B*_*y*_ and *B*_*z*_ being almost equal in value and *B*_*x*_ having spin-electric coupling values ca. 2 orders of magnitude smaller. However, when the electric field is oriented parallel to the pseudo-*C*_2_ axis (*y*), the magnetic field orientation dependence changes, with *B*_*y*_ now generating the smallest spin-electric coupling by approximately 2 orders of magnitude. We note that the magnitude of the coupling |⟨1_±_^′^|***H***_*E⃗*_|1_∓_^′^⟩| varies linearly with increasing electric field for *E*_*x*_ and *E*_*z*_. As a separate test, applying an external electric field only in the CASSCF-SO calculation, which has no impact on the molecular geometry and only on the electronic wave function, gives near zero spin-electric coupling. This confirms that the off-diagonal matrix elements arise from structural distortions induced by the applied electric field.

To understand the origin of the trends discussed above as a function of electric and magnetic field orientations, we have derived a perturbative expression for the spin-electric coupling within the ground doublet. Depending on the direction of *B⃗*, the Zeeman interaction rotates the two equilibrium crystal field eigenstates |1⟩ and |1̅⟩ within the ground Kramers doublet at energy *E*_1_ into new states |1_±_⟩. To linear order in |*B⃗*|, these states split in energy and mix with doublets at higher energies *E*_*m*_, giving rise to the perturbed ground doublet |1_±_^′^⟩, which is no longer symmetric under time reversal. The spin-electric coupling within the perturbed ground doublet is given by
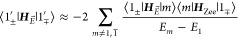
6where the sum runs over all the crystal field eigenstates |*m*⟩ belonging to higher energy doublets (see Section S2 for details). Thus, the most important contribution to the spin-electric coupling comes from the interdoublet mixing amplitudes ⟨1_±_ |***H***_*E⃗*_|*m*⟩ and ⟨*m*|***H***_Zee_|1_∓_⟩ of a few low lying doublets, owing to the energy denominator. The strong dependence on the Zeeman matrix element is illustrated in Table 1, which shows that states |2⟩, |2̅⟩ contribute at least 10 times more than any other excited doublet.

**Table 1:** Decomposition of the Spin-Electric Coupling |⟨1_±_^′^ |***H***_*E⃗*_ |1_∓_^′^⟩| from Equation 6 into the Contribution of Electric Field and Zeeman Mixing Amplitudes with Higher Lying States |*m*⟩^a^

state	contribution (%)	|⟨1 _±_ |***H***_*E⃗*_|*m*⟩| (cm^–1^)	|⟨1 _±_ |***H***_Zee_|*m*⟩| (cm^–1^)	*E*_*m*_ – *E*_1_ (cm^–1^)
|2⟩	74.505	5.726 × 10^1^	2.703 × 10^–1^	5.049 × 10^2^
|2̅⟩	21.691	1.986 × 10^1^	2.269 × 10^–1^	5.049 × 10^2^
|3⟩	2.061	3.409 × 10^1^	2.878 × 10^–2^	1.157 × 10^3^
|3̅⟩	1.712	2.632 × 10^1^	3.096 × 10^–2^	1.157 × 10^3^
|4⟩	0.019	6.041	2.616 × 10^–3^	2.028 × 10^3^
|4̅⟩	0.012	1.954	5.149 × 10^–3^	2.028 × 10^3^

a The electric and magnetic field strengths and orientations are *E_z_* = 3 × 10^9^ V m^–1^ and *B_z_* = 320 mT.

This perturbation expression gives good agreement with our ab initio results for *E*_*x*_ and *E*_*z*_ correctly predicting the magnetic field orientation dependence of the spin-electric coupling and gives good agreement with the spin-electric couplings from the electric field model (Figure S6; Section S2). Moreover, the perturbation expansion highlights the directional dependence of the spin-electric coupling through the decomposition into electric and Zeeman mixing amplitudes in the equilibrium geometry crystal field eigenbasis. First, we observe that the magnetic anisotropy of the Kramers doublets at energies *E*_*m*_ goes from rhombic for *m* = 1 to easy-axis for *m* ≥ 2, with the main anisotropy axis parallel to *x*, consistent with the axial disposition of the ligands (Table S2). This means that the crystal field eigenstates are close to eigenstates of ***J***_*x*_. As a consequence, applying a magnetic field along *x* only causes energy shifts, whereas *B⃗* fields along *y* and *z* cause significant interdoublet mixing (Figure S7b). According to our perturbation expression (eq 6), we then expect much smaller spin-electric couplings for *B*_*x*_ than for *B*_*y*_ and *B*_*z*_. This trend can be clearly observed for *E*_*x*_ and *E*_*z*_ (Figure 2c).

Using similar arguments, perturbation theory can be used to explain the electric field orientation dependence. When *E⃗* is oriented along the electric dipole moment (*y*), ***H***_*E⃗*_ becomes mostly diagonal (Figure S7a), which in turn means that the electric mixing amplitudes in eq 6 become smaller. This explains why the spin-electric coupling is smaller on average when *E⃗* is oriented along *y*, compared to other orientations (Figure 2c). We defer further details to Supporting Section S2.

**Figure 3:**
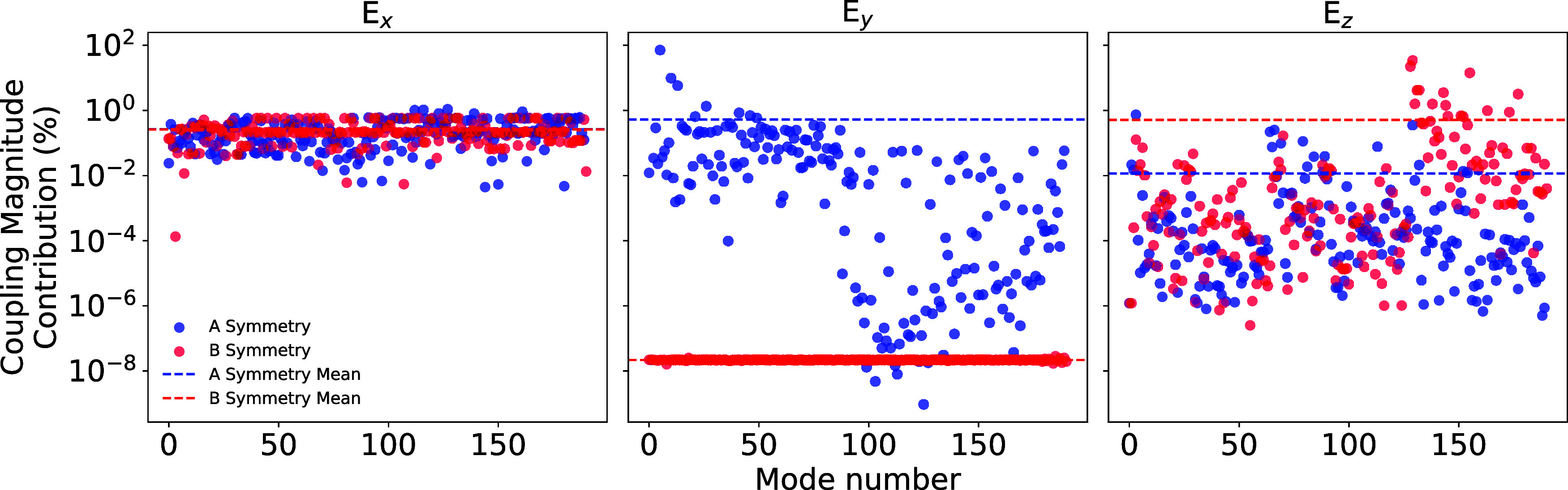
Contribution, which is defined as the spin-electric coupling value for a given mode (*A* or *B*) divided by the total spin-electric coupling of all modes, to the total spin-electric coupling magnitude due to an applied electric field along each Cartesian orientation (3 × 10^9^ V m^–1^) between the ground Kramers doublet split by the Zeeman effect due to a magnetic field aligned along Cartesian *z* (320 mT) for each “*A*” symmetry mode and each “*B*” symmetry mode in the symmetry adapted coordinate basis for the pseudo-*C*_2_ point group.

To further our understanding of the effect that the symmetry of electric field-induced distortions have on the spin-electric coupling, we can perform a pseudo-symmetry decomposition of the distorted molecular geometry in the vibrational normal mode basis in the *C*_2_ point group and extract the magnitude of the spin-electric coupling for each mode (Figure 3); see Supporting Information (Section S4) for details. When the electric field is perpendicular to the *C*_2_ axis, both *B* symmetry and *A* symmetry distortion modes are activated, giving mean contributions to the total spin-electric coupling of 0.513 and 0.01%, respectively, for |*E⃗*| = 3 × 10^9^ V m^–1^. In contrast, when the electric field is parallel to the pseudo-*C*_2_ axis, the *B* symmetry modes are suppressed (3.36 × 10^–9^%) and the *A* symmetry contributions dominate (0.53%); the suppression of *B* symmetry distortions leads to significantly smaller coupling. This allows us to rationalize that large spin-electric coupling is generated by distortions that break the equilibrium symmetry (Figure S7a). Hence, candidate molecules should be designed with a dipole moment oriented perpendicular to the high (pseudo-)symmetry axis such that the main distortions can break the (pseudo-)symmetry of the molecule.

### 3.2 Computational Electric Field Model

As DFT geometry optimizations with explicit electric fields are time-consuming and impose limits on the smallest field magnitude achievable, we have developed an analytical model based on a linear expansion in the electric field. Taking inspiration from Liu et al.,^23^ the idea is to approximate molecular distortions due to an applied electric field by equating the force due to the molecular potential energy , to the force exerted on the molecule by a uniform electric field in the electric dipole limit ***F*** = ∇_*r⃗*_μ⃗·*E⃗*, which is derived as the negative gradient of the potential energy due to an applied electric field *V*_*E⃗*_ = −μ⃗·*E⃗*. The structural distortion *r⃗* is obtained by solving the following set of linear equations:
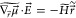
7where *r⃗* is the set of 3*N* atomic displacements due to the applied electric field, *E⃗* is the three component electric field vector, ∇_*r⃗*_μ⃗ is the derivative of the molecular dipole moment with respect to atomic displacements *r⃗* (n.b. this is not the polarizability), and ***H*** is the Hessian matrix. The tilde notation denotes that the matrix has been reduced from 3*N* normal mode displacements to 3*N* – *k* normal mode displacement (*k* = 5, 6) in which energy invariant rigid body rotations and translations have been projected out as they do not contribute to geometric distortions.

It is worth stressing at this point that the presence of a permanent electric dipole μ⃗ ≠ 0 (i.e., broken spatial inversion symmetry) is not strictly necessary for generating a spin-electric coupling. In fact, even if the N–Tm–N bond was perfectly linear with *μ⃗* = 0, an electric field would distort the structure owing to the delocalized character of the molecular charge density. Despite including only the electric dipole in our model and ignoring all higher multipole terms, which represent the finite extent of the molecular charge density, our model still captures this effect. In fact, as shown in eq 7, the relevant quantity for determining structural distortions to leading order in the electric field strength is the electric dipole gradient with respect to the nuclear positions, ∇_*r⃗*_μ⃗.

A full derivation and details of our implementation are in S1. The workflow for predicting spin-electric couplings using the electric field model combined with the LVC and the inherent savings in computational expense is represented in graphical (Figure S5).^55^ This analytic model only requires a single geometry optimization and frequency calculation in the gas phase under zero electric field, from which the Hessian and the electric dipole derivatives are obtained. Coupled with a single-shot ab initio approach for obtaining derivatives of the Hamiltonian as a function of molecular coordinates using an LVC model that utilizes the first order derivatives of the Hamiltonian matrix elements, this method offers considerable benefits in computational expense.^55^

**Figure 4:**
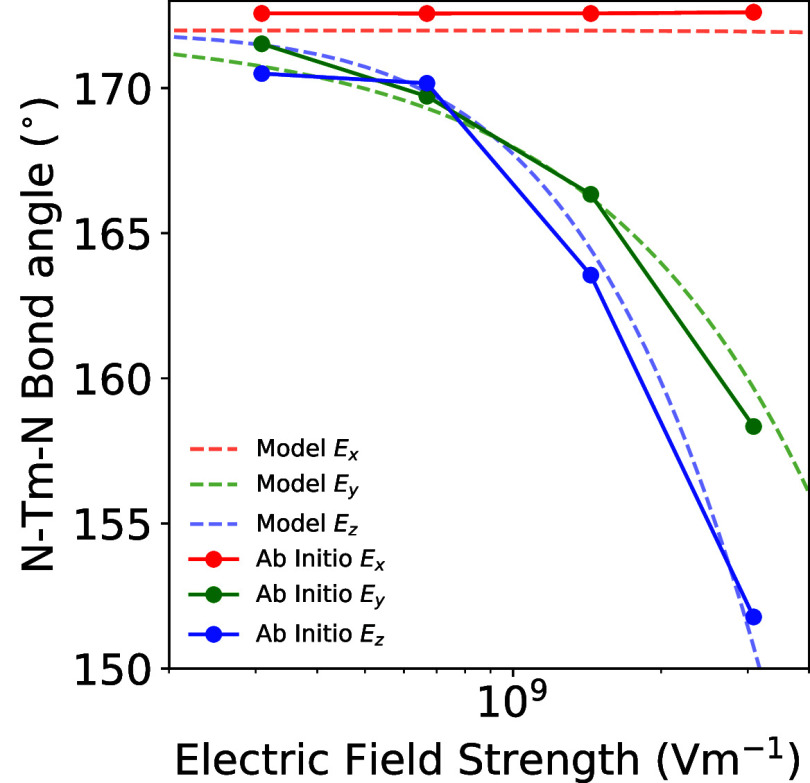
Comparison of the bond angle of the main N–Tm–N molecular axis in Tm(N^††^)_2_ between pure ab initio electric field optimization in Gaussian 16 and the vibronic model for electric field magnitudes spanning an order of magnitude for each Cartesian direction.

**Figure 5:**
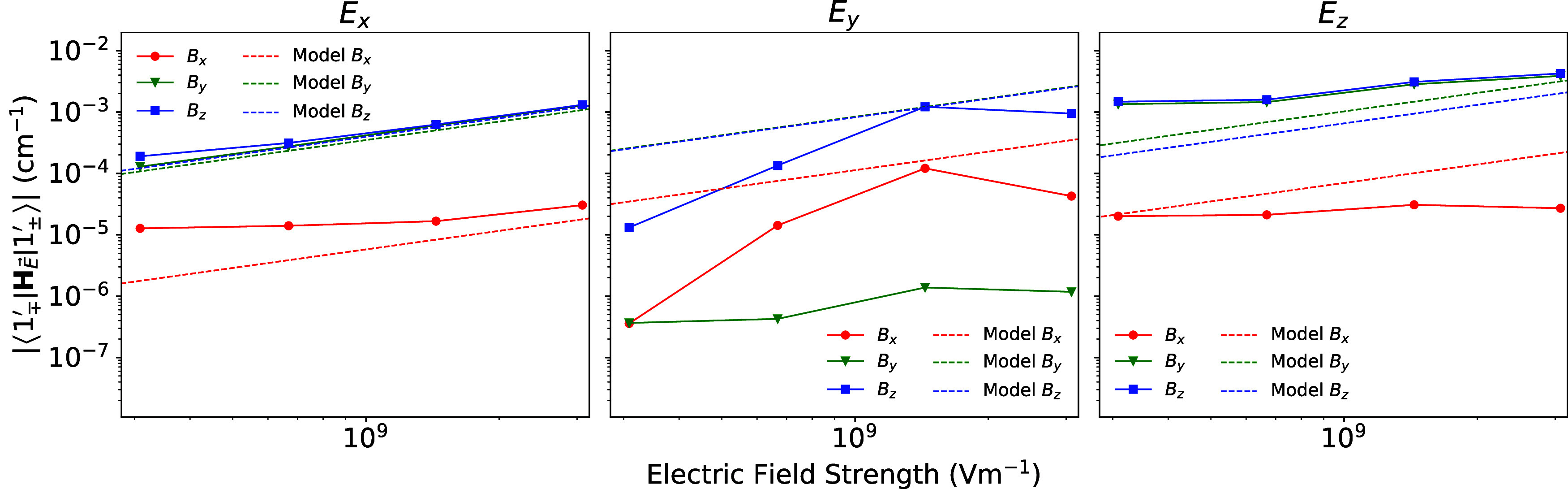
Comparison between the spin-electric couplings calculated from explicit ab initio geometry optimizations with an applied electric field and CASSCF calculations for each geometry against the spin-electric couplings calculated from distorted geometries predicted by our analytical model and the equilibrium geometry parametrized LVC model. For each relative orientation of the magnetic and electric fields of strengths 320 mT and (10^8^ – 10^10^ V m^–1^).

Our analytic model shows excellent agreement with ab initio when comparing the N–Tm–N bond angle as a function of field strength (Figure 4). Furthermore, the agreement of this model with explicit ab initio electric field optimization is further shown when comparing the root-mean-square deviation (RMSD) of distorted geometries to the equilibrium structure as a function of field strength (Figure S1). Hence, we are able to investigate smaller electric fields like those employed for experiments on molecular qubits which use electric field strengths on the order of 10^6^ V m^–1^.^24^ The spin-electric couplings calculated using this approach show very good agreement in the coupling magnitudes and the magnetic and electric field orientation dependence for *E*_*x*_ and *E*_*z*_ (Figure 5). The agreement is further supported when the spin-electric coupling for all electric field orientations is plotted as an isosurface (Figure S3). However, there are discrepancies between our model’s prediction and the results obtained from explicit ab initio electric field optimizations: when the electric field is oriented parallel to the high (pseudo-)symmetry *C*_2_ axis *y*, with our model predicting a much larger effect. This arises because the distorted geometry produced by our electric field model does not preserve the pseudo-*C*_2_ symmetry, while the explicit ab initio electric field optimization does. As a result, the symmetry breaking distortions that generate the spin-electric coupling are enhanced in our model, leading to increased spin-electric coupling (Figure S2). However, the magnetic orientation dependence is also different using the model when *E⃗* is directed along *y*, as its predictions of the magnetic field orientation dependence are consistent with those of symmetry breaking distortions in *E*_*x*_ and *E*_*z*_; although this might actually be expected given the perturbation expression described by eq 6.

The ability of our simple linear model to capture the behavior of the spin-electric coupling over a range of electric field magnitudes and orientations, demonstrates its potential as an efficient methodology to explore molecular spin-electric coupling and to determine the optimal electric field orientation.

### 3.3 Electric Field Driven Spin Dynamics

**Figure 6:**
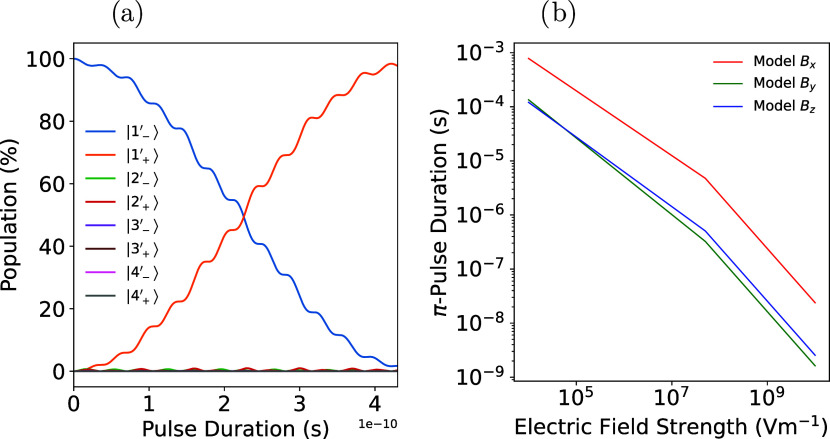
(a) The population of states |*n*_±_^′^⟩ as a function of pulse duration using the dynamics described in eq 8 where *E*_*z*_ = 3 × 10^9^ V m^–1^, *B*_*z*_ = 320 mT, and a driving frequency of 14.2 GHz. (b) The pulse duration required to perform a π-pulse between the ground doublet states |1_±_^′^⟩ calculated using the spin-electric couplings obtained from our model.

Simulation of an effective π-pulse between the ground doublet states |1_–_^′^⟩ and |1_+_^′^⟩ using a resonant frequency electric field pulse was performed using the Liouville-Von Neumann equation, where the spin density matrix **ρ** is initialized with all spin population in state |1_–_^′^⟩. The dynamics is driven using the electric field Hamiltonian oscillating at frequency ω which is given by the splitting of the ground doublet due to the weak static magnetic field shown in eq 8, we defer further details of the derivation and implementation to Section S3.
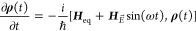
8Our simulation of the spin dynamics (Figure 6a) shows complete population transfer between the ground doublet states with a pulse duration of 0.4 ns for a field strength of 3 × 10^9^ V m^–1^. Our simulation shows good agreement with the pulse duration predicted from the Rabi frequency calculated from ab initio spin-electric couplings (Figure S8a). Using the results from our spin-electric coupling model, the π-pulse duration for a field of 10^6^ V m^–1^ is of the order of microseconds (Figure 6b). The requirement of driving the transition with resonant frequency pulse is illustrated by calculating the transition probability (|*c*(*t*)|^2^) as a function of pulse duration and electric field drive detuning (Figure S8c). As the driving frequency of the electric field is detuned further from resonance to completely off resonance the transition probability decreases to ≈0. Hence, a static electric field would be unable to drive the transition of spin-population in a commensurate time scale to spin decoherence.

### 3.4 Coordination Geometry and Point Group Symmetry

**Figure 7:**
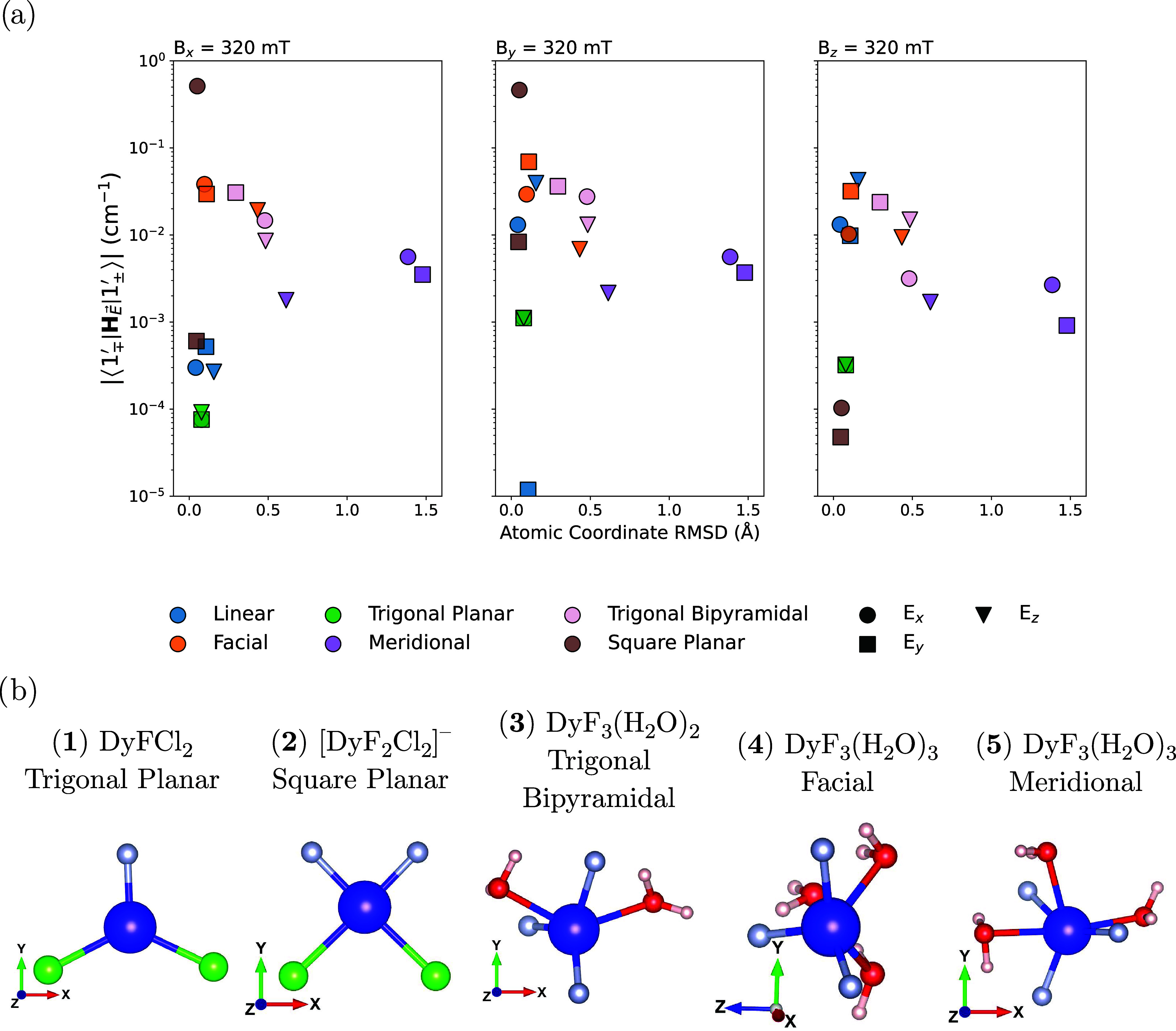
(a) Spin-electric coupling for a range of “toy” lanthanide molecules, with varying coordination geometries and point group symmetries, plotted against the RMSD of the distortion due to an applied electric field, for each Cartesian orientation of the electric field of strength 3 × 10^9^ V m^–1^, for each magnetic field orientation of strength 320 mT. (b) A visual representation of the geometry optimized “toy” molecules where the atoms are represented as: Dy, Blue; F, Silver; Cl, Green; O, Red; H, White. Structure visualization was performed using VESTA.^58^

Following our investigations into Tm(N^††^)_2_, we were curious how different coordination geometries may exhibit larger electric field-induced symmetry breaking and hence stronger spin-electric coupling. To assess this question, we designed a range of toy molecules spanning a range of coordination geometries including trigonal planar DyFCl_2_ (**1**), square planar [DyF_2_Cl_2_]^−^ (**2**), trigonal bipyramidal DyF_3_(H_2_O)_2_ (**3**), and the facial and meridional octahedral isomers of DyF_3_(H_2_O)_3_ (**4, 5**) shown in Figure 7b. Each molecule was designed with a nonzero molecular dipole moment to ensure an electric field dependence. To quantify the extent of molecular distortion under an applied field we assess the RMSD of the resulting structures. However, just as observed for Tm(N^††^)_2_, we find that the structural RMSD has little bearing on the spin-electric coupling: for an electric field strength of 3 × 10^9^ V m^–1^, mer-DyF_3_(H_2_O)_3_ (**5**) shows the largest structural RMSD (ca. 1.5 Å) yet has relatively small spin-electric coupling magnitudes (ca. 10^–3^ cm^–1^), compared to the largest observed spin electric couplings of ca. 10^–1^ cm^–1^ for fac-DyF_3_(H_2_O)_3_ (**4**) which shows much smaller structural distortions of RMSD ca. 0.4 Å. Indeed across all geometries tested here there is no correlation between structural RMSD and the spin-electric coupling (Figure 7a). Interestingly, fac-DyF_3_(H_2_O)_3_, which has the largest spin-electric coupling among the isomers considered, demonstrates almost no electric or magnetic field orientation dependence. The lack of electric field orientation dependence can be rationalized as fac-DyF_3_(H_2_O)_3_ has a nonzero dipole moment in all orientations of the electric field, hence structural distortions that break the pseudo-*C*_3_ symmetry can occur with any electric field orientation. The lack of magnetic field orientation dependence can be understood because neither the ground nor the excited states are well-approximated as eigenstates of any Cartesian projection of the total angular momentum. Similarly, trigonal planar DyFCl_2_ (**1**) (*C*_2*v*_) has no electric field orientation dependence however it shows only small spin-electric couplings (ca. 10^–3^–10^–2^ cm^–1^). In this case, when the electric field is in along the *C*_2_ axis (*y*) the distortion is symmetry preserving so no extra mixing is induced. When the electric field is oriented in the molecular plane but perpendicular to the high-symmetry axis, there is a near-zero dipole moment so the effect is minor. When the electric field is perpendicular to the molecular plane (*z*) this breaks the *C*_2_ symmetry but preserves the σ_v_ mirror plane and such yields only small spin-electric coupling. After optimization, the trigonal bipyramidal DyF_3_(H_2_O)_2_ (**3**) has *C*_2_ symmetry like Tm(N^††^)_2_, however we do not observe the same electric field orientation dependence as for Tm(N^††^)_2_ because the electric fields were not oriented parallel to the high symmetry axis and therefore all distortions are symmetry breaking and the couplings are large (ca. 10^–2^ cm^–1^). The largest spin-electric coupling for the toy models was generated by an electric field applied perpendicular to the high-symmetry *C*_2_ axis of the square planar toy geometry ([DyCl_2_F_2_]^−^). The square planar geometry (**2**) at equilibrium has a *C*_2_ axis and two σ_v_ mirror planes, and for an electric field (3 × 10^9^ V m^–1^) applied perpendicular to the high-symmetry axis in the molecular plane (*x*) we see the breaking of the *C*_2_ axis and one of the σ_v_ mirror planes due to a reduction in one of the Dy–Cl bonds from 2.67 Å to 2.58 Å. The breaking of both the high-symmetry *C*_2_ axis and a σ_v_ mirror plane leads to a significant lowering of point-group symmetry and therefore a large spin-electric coupling (6.53 × 10^–1^ cm^–1^). The square planar model shows its smallest spin-electric coupling when the electric field is oriented along *z* which arises from the strong axial anisotropy oriented along *z* of excited Kramers doublets.

Our findings from investigating “toy” molecular geometries further support our conclusions that good candidate molecules should be designed to have high (pseudo-)symmetry and have dipole moments perpendicular to the (pseudo-)symmetry axis.

## 4 Conclusions

We have unveiled the underlying principles that generate spin-electric coupling in molecular qubits through the use and analysis of ab initio simulations on [Tm(N(Si^i^Pr_3_)_2_)_2_] and a range of simple lanthanide molecules with varying coordination geometries and symmetries. We have proposed an analytic model to obtain distorted molecular geometries for electric fields of experimentally relevant magnitude with any orientation, which in conjunction with a linear vibronic coupling model, allows us to directly obtain the perturbing Hamiltonian due to the electric field. Hence, from an optimized molecular geometry and vibrational mode calculation, and a single-shot CASSCF-SO calculation of the electronic structure, our methods can directly yield the spin-electric coupling. Using density matrix spin dynamics we have demonstrated a proof-of-concept coherent manipulation of a molecular spin in our *S̃* = 1/2 system using a resonant electric field pulse. We have outlined a computational method to make predictions of the driving frequency and pulse duration required to perform molecular spin manipulation using electric fields in an experimental setting. Summarizing our findings, we suggest the following guidelines for designing molecular qubits with large spin-electric couplings: (i) a metal center with large unquenched orbital angular momentum, for which lanthanide ions are the obvious choice; (ii) high molecular (pseudo-)symmetry, for two reasons (a) large derivatives of the electric dipole moment with respect to nuclear coordinates, ∇_*r⃗*_μ⃗, are required to maximize the effect of an applied electric field, and these are likely to arise as molecular symmetry is broken, and (b) large spin-electric coupling arises from symmetry breaking distortions, and molecules with high-(pseudo-)symmetry have better prospects of symmetry breaking; (iii) the applied electric field should be oriented perpendicular to the high-(pseudo-)symmetry molecular axis, so that the electric-field-induced distortion breaks the (pseudo-)symmetry, to maximize mixing between the ground and excited Kramers doublets; and (iv) the bias magnetic field should be perpendicular to the magnetic anisotropy axis of the first excited Kramers doublets to maximize mixing between ground and excited states. While it may be nontrivial to realize all of these features in a single molecule, this is the first proposals for design criteria that can now be tested for viability and effect.

## Data Availability

Research data can be found at doi: 10.48420/26617561.

## Supporting Information Available

Full derivation of our electric field model and information on its derivations; a full derivation of the perturbative expression with a detailed explanation of the electric and magnetic field orientation dependence; comparisons of our perturbative expression to ab initio and our electric field model calculated spin-electric coupling; details on our implementation of our electric field driven spin dynamics and its limitations; a derivation of our pseudo-symmetry decomposition of molecular distortions and its implementation; the decomposition of the crystal field wave function of the equilibrium geometry; and a table containing the exact calculated spin-electric coupling for each orientation of the electric and magnetic fields (PDF)
