# Correction to “Ab Initio Design of Molecular Qubits with Electric Field Control”

**DOI:** 10.1021/jacs.5c08471

**Published:** 2025-06-20

**Authors:** William T. Morrillo, Herbert I. J. Cumming, Andrea Mattioni, Jakob K. Staab, Nicholas F. Chilton

Our work used computational methods to study control of electron spins using applied electric fields.^1^ After we determine the molecular distortion due to an applied electric field, we approximate the electronic Hamiltonian using a linear vibronic coupling (LVC) model. The first step of this approximation is to map the distorted geometry onto the equilibrium geometry where the LVC model was parametrized in order to minimize the Euclidean distance between the molecular structures and hence minimize the truncation error of the LVC approximation. However, the implementation of the Kabsch algorithm^2^ used to align molecular geometries was incorrect, giving an incorrect molecular rotation. Hence, the resulting crystal field parameters (*B*_*k*_^*q*^) obtained from evaluating electric field distorted geometries using the LVC were inaccurate. The error in the crystal field parameters increases as the distortion of the molecule grows, resulting in large errors for geometries at high electric fields. The alignment error also resulted in an effective change of reference frame away from the molecular reference frame. In-turn, our magnetic fields were no longer oriented along the molecular axes thereby affecting the magnetic field orientation dependence of the spin-electric coupling in Figure 5. We also found a small error in the *ab initio* spin-electric coupling in Figures 2c and 5, where the spin-electric coupling was 1 order of magnitude too small due to being calculated using a magnetic field strength of 32 mT rather than 320 mT. In this correction Figure 1 replaces Figure 2c in the original work, while Figures 2 and 3 replace Figures 3 and 5, respectively.

**Figure 1:**
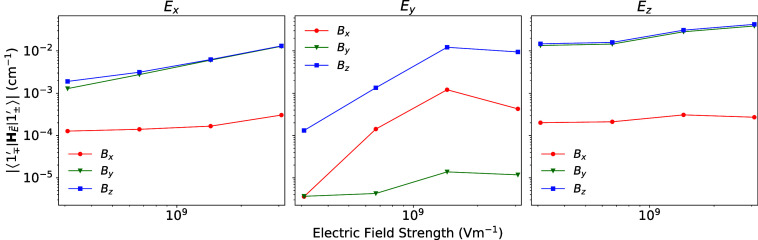
Orientation dependence of the coupling element between the ground double states split by an applied magnetic field in each Cartesian direction for each electric field orientation.

**Figure 2:**
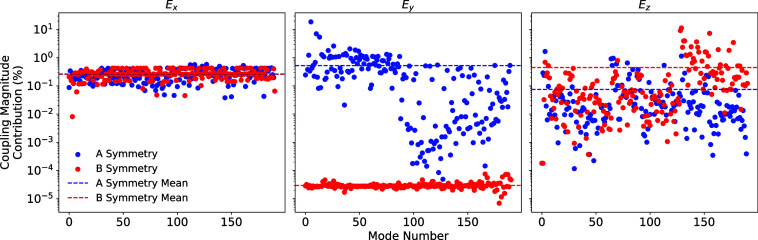
Contribution, which is defined as the spin-electric coupling value for a given mode (*A* or *B*) divided by the total spin-electric coupling of all modes, to the total spin-electric coupling magnitude due to an applied electric field along each Cartesian orientation (3 × 10^9^ V m^–1^) between the ground Kramers doublet split by the Zeeman effect due to a magnetic field aligned along Cartesian *z* (320 mT) for each “*A*” symmetry mode and each “*B*” symmetry mode in the symmetry adapted coordinate basis for the pseudo-C_2_ point group.

**Figure 3:**
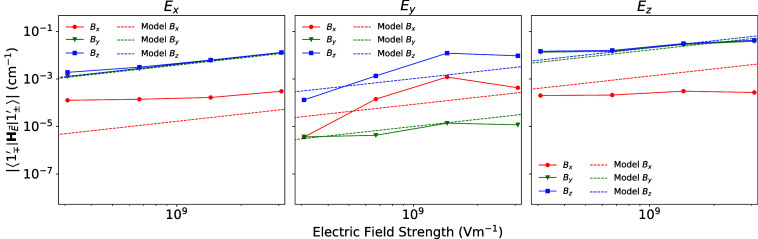
Comparison between the spin-electric couplings calculated from explicit *ab initio* geometry optimizations with an applied electric field and CASSCF calculations for each geometry against the spin-electric couplings calculated from distorted geometries predicted by our analytical model and the equilibrium geometry parametrized LVC model for each relative orientation of the magnetic and electric fields of strengths 320 mT and 10^8^–10^10^ V m^–1^.

In the original work, we believed that the discrepancy in the spin-electric coupling between the electric field model and *ab initio* simulations when the electric field was oriented along the *y*-axis was attributable to the model’s inability to replicate the symmetry-preserving behavior we observed in our *ab initio* findings. However, with the corrected rotation here, the electric field model accurately predicts the magnetic field orientation dependence for all electric field orientations (Figure 3). Our conclusions surrounding the electric field model has not changed, and in fact, we now see improved agreement with the electric field model; it is more robust than we originally thought.

The data in the associated repository (doi: 10.48420/26617561) and the code implicated (spin_phonon_suite) have been updated.

## Supplementary Material



## Notes

Full derivation of our electric field model and information on its derivations; a full derivation of the perturbative expression with a detailed explanation of the electric and magnetic field orientation dependence; comparisons of our perturbative expression to *ab initio* and our electric field model calculated spin-electric coupling; details on our implementation of our electric field driven spin dynamics and its limitations; a derivation of our pseudo-symmetry decomposition of molecular distortions and its implementation; the decomposition of the crystal field wave function of the equilibrium geometry; table containing the exact calculated spin-electric coupling for each orientation of the electric and magnetic fields (PDF)
