# Supplementary material for: Ab Initio Design of Molecular Qubits with Electric Field Control
Source: J Am Chem Soc. 2024 Sep 5;146(37):25841–51. doi: 10.1021/jacs.4c09109 (PMC11421027; doi:10.1021/jacs.4c09109)
Supplement: Supplementary file 1 — ja4c09109_si_001.pdf [file ja4c09109_si_001.pdf]

Supporting Information

Ab Initio Design of Molecular Qubits with Electric Field Control

William T. Morrillo *[1], Herbert I. J. Cumming[1], Andrea Mattioni[1], Jakob K. Staab[1], and Nicholas F. Chilton †[1,2]

[1]Department of Chemistry, The University of Manchester, Oxford Road, Manchester, M13 9PL, UK.

[2]Research School of Chemistry, The Australian National University, Canberra, 2601, Australia.

Contents

S1 Analytic Model **2**

S2 Perturbation Theory Derivation **9**

S3 Spin dynamics driven with resonant frequency electric fields. **16**

S4 Pseudo C_2 Irrep Symmetry Decomposition **18**

S5 Electronic Structure **20**

S6 *Ab Initio* Spin-electric Couplings **21**

*william.morrillo@manchester.ac.uk
†nicholas.chilton@anu.edu.au

S1 Analytic Model

Due to limitations in both computational capability and cost the need for an analytical model of electric field distortions is required to aid in high throughput calculations and in calculations for electric field strengths smaller than the smallest available electric field *ab initio* ($1 \times 10^8\,\mathrm{V\,m^{-1}}$) to replicate experimental results.

We first define the potential energy of the system to be given as:

$$V = \frac{1}{2}\vec{r}^T \boldsymbol{H}\vec{r} + \underbrace{\vec{g}\cdot\vec{r}}_{\boldsymbol{g}=0} \tag{S1}$$

Where $\vec{r}$ is the atomic coordinate vector containing 3N entries, $\boldsymbol{H}$ is the Hessian matrix obtained from a frequency calculation at the equilibrium geometry and $\vec{g}$ is the potential energy gradient vector which can be assumed to be zero at the equilibrium geometry. The force associated with the displacement of atoms is given as the negative gradient of the potential energy and is given by:

$$\boldsymbol{F} = -\nabla_{\vec{r}} V \tag{S2}$$

To compute the gradient of the potential energy with respect to atomic coordinate displacements, equation S1 is written in terms of its components.

$$V = \frac{1}{2}\sum_{ij} r_i H_{ij} r_j \tag{S3}$$

$$V = \frac{1}{2}\sum_{i}\left(H_{ii}r_i^2 + \sum_{j\neq i} r_i H_{ij} r_j\right) \tag{S4}$$

Where terms the same indices are taken outside of the summation. Taking the derivative the potential with respect to a general element k we get.

$$\frac{\partial V}{\partial r_k} = \frac{1}{2}\left(2H_{kk}r_k + \sum_{i\neq k} r_i H_{ik} + \sum_{j\neq k} H_{kj} r_j\right) \tag{S5}$$

Adding the kth element of the derivative back into the summations we get.

$$\frac{\partial V}{\partial r_k} = \frac{1}{2}\left(\sum_i r_i H_{ik} + \sum_j H_{kj} r_j\right) \tag{S6}$$

Writing equation (S6) back into matrix form gives the following equations.

$$\nabla_{\vec{r}} V = \frac{1}{2}\begin{bmatrix} \sum_i^l r_i H_{i1} + \sum_j^l H_{1j} r_j \\ \sum_i^l r_i H_{i2} + \sum_j^l H_{2j} r_j \\ \vdots \\ \sum_i^l r_i H_{il} + \sum_j^l H_{lj} r_j \end{bmatrix} = \frac{1}{2}\begin{bmatrix} \sum_i^l r_i H_{i1} \\ \sum_i^l r_i H_{i2} \\ \vdots \\ \sum_i^l r_i H_{il} \end{bmatrix} + \frac{1}{2}\begin{bmatrix} \sum_j^l H_{1j} r_j \\ \sum_j^l H_{2j} r_j \\ \vdots \\ \sum_j^l H_{lj} r_j \end{bmatrix} \tag{S7}$$

$$\nabla_{\vec{r}} V = \frac{1}{2}\boldsymbol{H}^T \vec{r} + \frac{1}{2}\boldsymbol{H}\vec{r} = \frac{1}{2}\left(\boldsymbol{H}^T + \boldsymbol{H}\right)\vec{r} \tag{S8}$$

Where l is the number of modes. The Hessian is a symmetric matrix ($\boldsymbol{H^T} = \boldsymbol{H}$) such that the gradient of the potential energy can be written as $\boldsymbol{H} \cdot \boldsymbol{r}$ as such the force is given by.

$$\boldsymbol{F} = -\nabla_{\vec{r}} V = -\boldsymbol{H} \cdot \vec{r} \tag{S9}$$

To calculate the distortion due to an applied electric field, we calcualate the potential energy due to the applied electric field and the associated force. The potential energy due to an applied electric field can be written as a power series expansion of the molecular potential energy as a function of electric field.

$$V = V_0 - \left(\vec{\mu}\vec{E} + \vec{\alpha}\frac{\vec{E}^2}{2} + \vec{\beta}\frac{\vec{E}^3}{6}\dots\right) \tag{S10}$$

Where V_0 is the molecular potential energy at zero field, $\boldsymbol{\mu}$ is the dipole moment, $\vec{\alpha}$ is the polarisability, $\vec{\beta}$ is the hyperpolarisability, and $\vec{E}$ is the electric field vector. Here we truncate the Taylor series expansion of the potential energy to fist order as the term that depends on the electric field squared has negligible contribution to structural distortions. Hence, we take the potential energy due to an electric field to be:

$$V = -\vec{\mu} \cdot \vec{E} \tag{S11}$$

The force exerted of the atomic coordinated due to an electric field, $\boldsymbol{F_E}$, is therefor given as the negative of gradient of the electric dipole moment with respect the the displacement of atomic coordinates.

$$\boldsymbol{F_E} = \nabla_{\vec{r}}\vec{\mu} \cdot \vec{E} \tag{S12}$$

Alternatively this can be written in terms of its components such that the αth component of the force exerted on atom i due to an applied electric field is given by:

$$\vec{F}_{i,a} = \frac{\partial \mu_\alpha}{\partial r_i} \cdot \vec{E} \tag{S13}$$

The atomic electric dipole derivatives are obtained from directly from the initial geometry optimisation maintaining the the single shot approach of this method. The force due to electric field and the force due to atomic displacements are set equal to each other giving the following total equation for the distortion due to an electric field.

$$\nabla_{\vec{r}}\vec{\mu} \cdot \vec{E} = -\boldsymbol{H} \cdot \vec{r} \tag{S14}$$

This forms a set of exact linear equations for $\vec{r}$. However, the the Hessian still contains the energy invariant rigid body translations and rotations (3 and 6 for the solid state and gas phase respectively) which should not be included when finding a solution. We construct a new orthonormal basis of internal coordinate displacement vectors $\boldsymbol{U}$ such they have dimensions $[3N \times 3N - k]$ where the energy invariant rigid body displacements are removed. We can then transform the set of linear equations into the new reduced rank $\boldsymbol{U}$ basis to form a new set of linear equations with the energy invariant rigid body rotations and translations projected out.

$$\boldsymbol{U}^T \nabla_{\vec{r}}\vec{\mu} \cdot \vec{E} = - \underbrace{\boldsymbol{U}^T\boldsymbol{H}\boldsymbol{U}}_{[3N-k\times 3N-k]} \boldsymbol{U}^T\vec{r} \tag{S15}$$

Where the solution to the set of linear equations is given by $\boldsymbol{U^T r}$, where $\boldsymbol{U^T}\vec{r}$ is the projection of $\vec{r}$ in to the column space of $\boldsymbol{U}$. Such that our full rank solution is projected

back into the atomic displacement vector space.

$$\vec{r} = \boldsymbol{U} \underbrace{\boldsymbol{U}^T \vec{r}}_{\text{Reduced rank solution}} \tag{S16}$$

Where $\boldsymbol{UU^T} = \mathbf{1}$ as $\boldsymbol{U}$ is an orthonormal basis. This yields the distortion vector $\boldsymbol{r}$.

The units of quantities extracted from Gaussian 16 can be somewhat ambiguous and therefore we provide a table of extracted units and the conversions we perform form the analytical model. All unit conversions are performed using the physical constants library

Table S1: All unit conversions from the extracted Gaussian16 quantities to the units used in the analytical model.

Quantity	Gaussian16 unit	Model unit
$\nabla_{\vec{r}}\vec{\mu}$	e	C
$\boldsymbol{H}$	$E_\mathrm{h}\, a_0^{-2}$	$\mathrm{J\,m^{-2}} = \mathrm{N\,m^{-1}}$
$\vec{r}_\mathrm{eq}$	a_0	Å
$\vec{E}$	$\mathrm{V\,m^{-1}}$	$\mathrm{V\,m^{-1}} = \mathrm{N\,C}$

as part of the scipy package for python.

$$-\boldsymbol{H} \cdot \vec{r} = \nabla_{\vec{r}}\vec{\mu} \cdot \vec{E} \tag{S17}$$

$$\frac{\mathrm{N\,m}}{\mathrm{m}} = \frac{\mathrm{C\,N}}{\mathrm{C}} \tag{S18}$$

The calculated displacements are then converted from m to Å to be added to the equilibrium coordinates extracted from the formatted checkpoint file.

Our electric field model can be used to obtain the derivatives of the crystal field Hamiltonian with respect to an applied electric field $\frac{\partial \boldsymbol{H}}{\partial \vec{E}}$ using the derivatives obtained from the LVC model. The electric field model gives structural distortions as a function of electric field strength. We use the autodiff library which is a part of the JAX library to obtain the analytical derivative of the structural distortion as a function of electric field strength $\frac{\partial \vec{r}}{\partial \vec{E}}$.

Using the derivatives of the crystal field Hamiltonian with respect to atomic coordi-

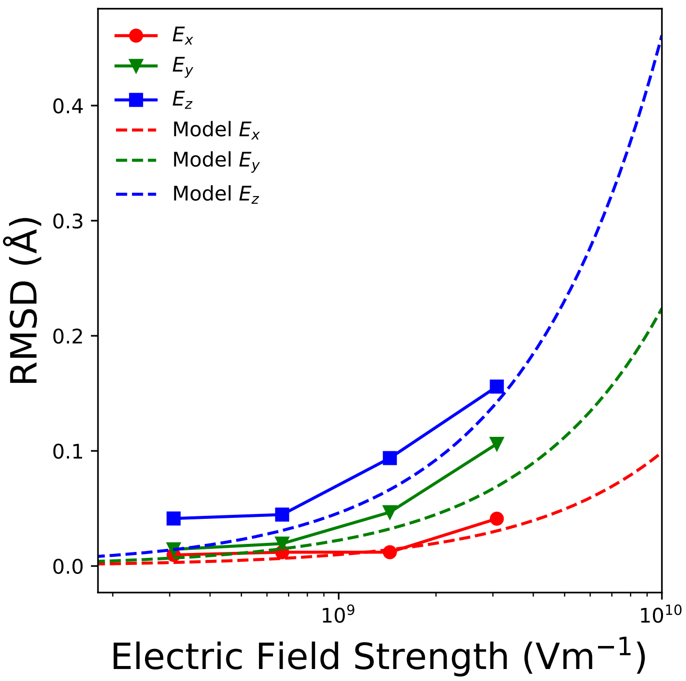

Figure S1: The atomic coordinate Root Mean Square Distance (RMSD) of the *ab initio* and electric field model geometries in comparison to the equilibrium geometry as a function of electric field strength.

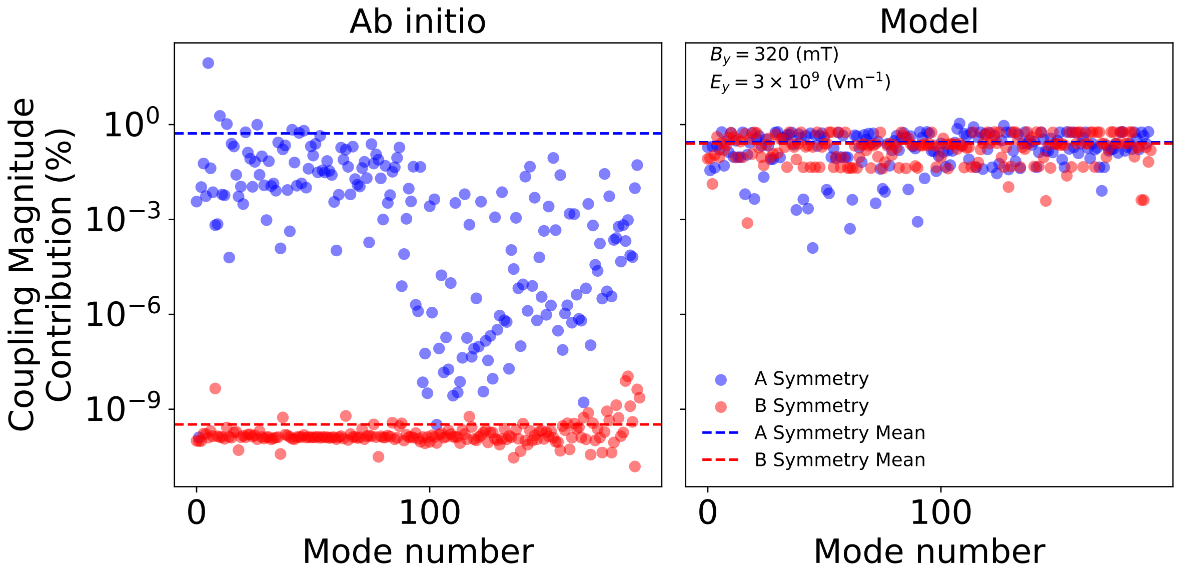

Figure S2: The decomposition of the *ab initio* and analytical model predicted structures into the symmetry adapted coordinated basis with the contribution each symmetry has to the total spin-electric coupling magnitude. For a magnetic field of $B_y = 320\,\mathrm{mT}$ and an electric field of $E_y = 3 \times 10^9\,\mathrm{V\,m^{-1}}$.

nates from the LVC model we obtain the derivative with respect to an applied electric field.

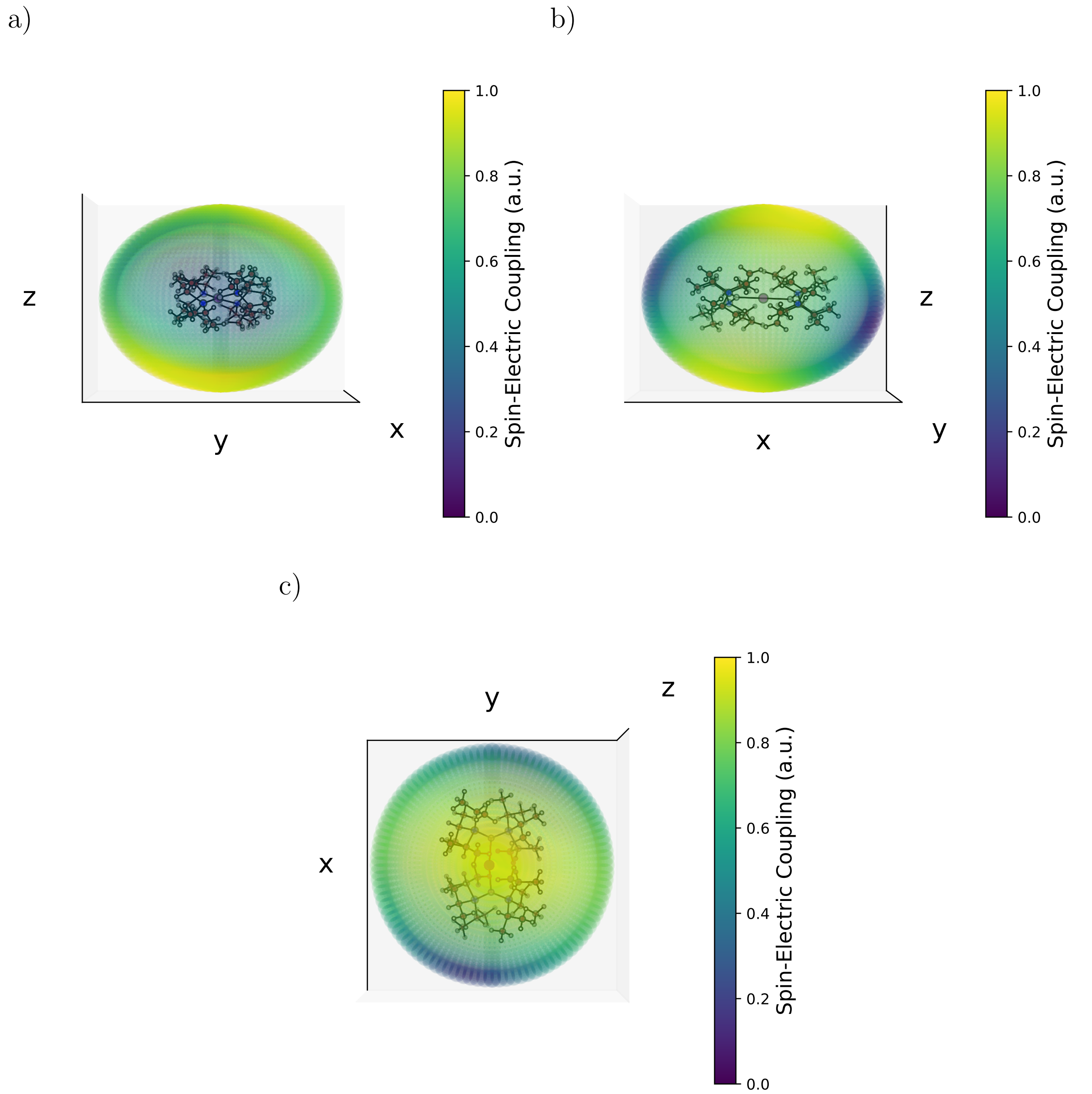

Figure S3: The normalised spin-electric coupling of $Tm(N^{\dagger\dagger})_2$ plot as an isosurface for all orientations of an electric field surrounding the molecular structure of the equilibrium geometry $Tm(N^{\dagger\dagger})_2$. a) Looking along the x axis. b) Looking along the y axis. c) looking along the z axis.

$$\frac{\partial H_{m,n}}{\partial E_\beta} = \sum_{i,\alpha} \frac{\partial H_{m,n}}{\partial r_{i,\alpha}} \cdot \frac{\partial r_{i,\alpha}}{\partial E_\beta} \tag{S19}$$

Using these analytical derivatives we can evaluate the electric field Hamiltonian for any given electric field without the requirement to evaluate the geometric distortion due to an applied electric field first.

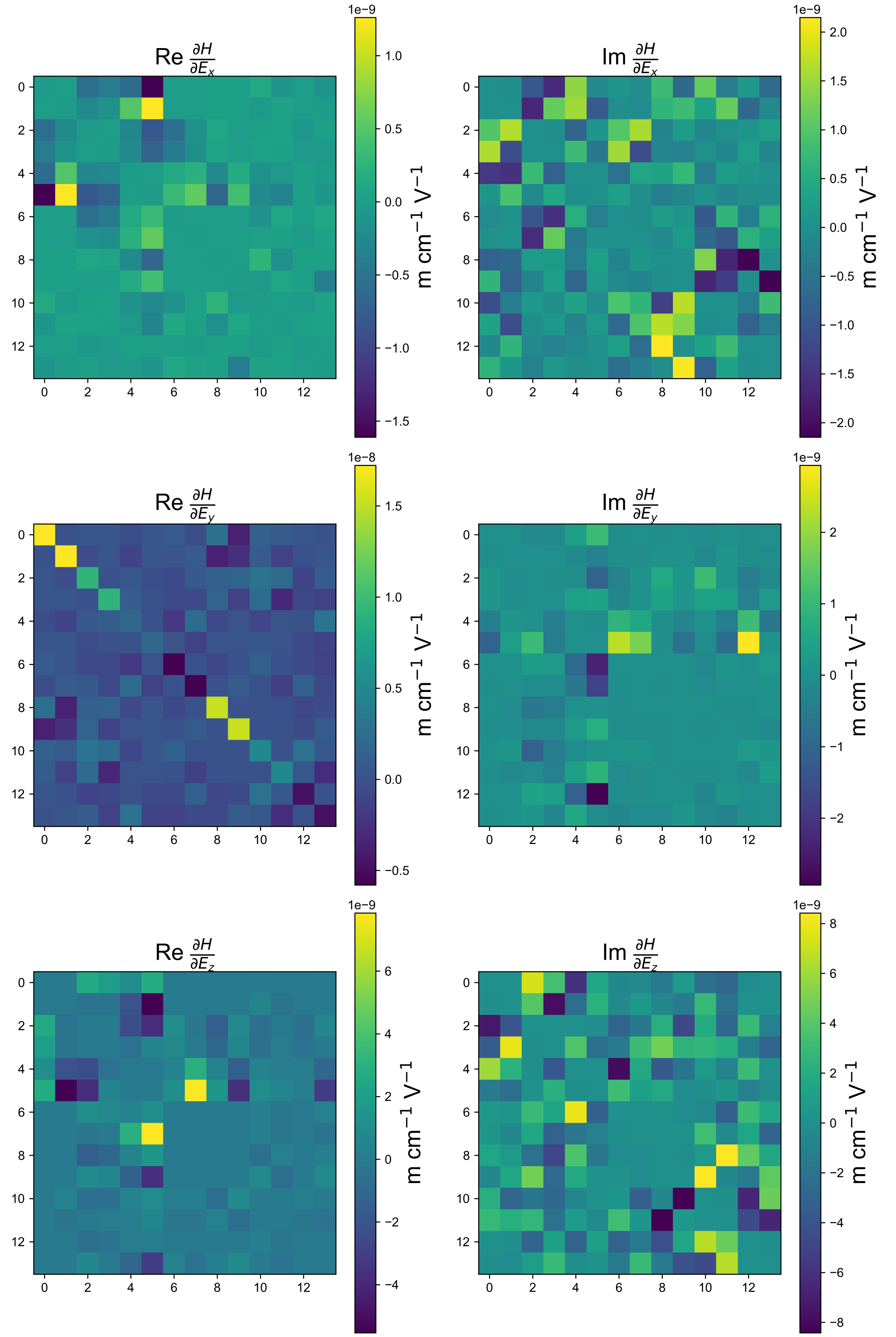

Figure S4: The real and imaginary derivatives of the distorted crystal field Hamiltonian in the SO basis for each electric field orientation (x, y, z).

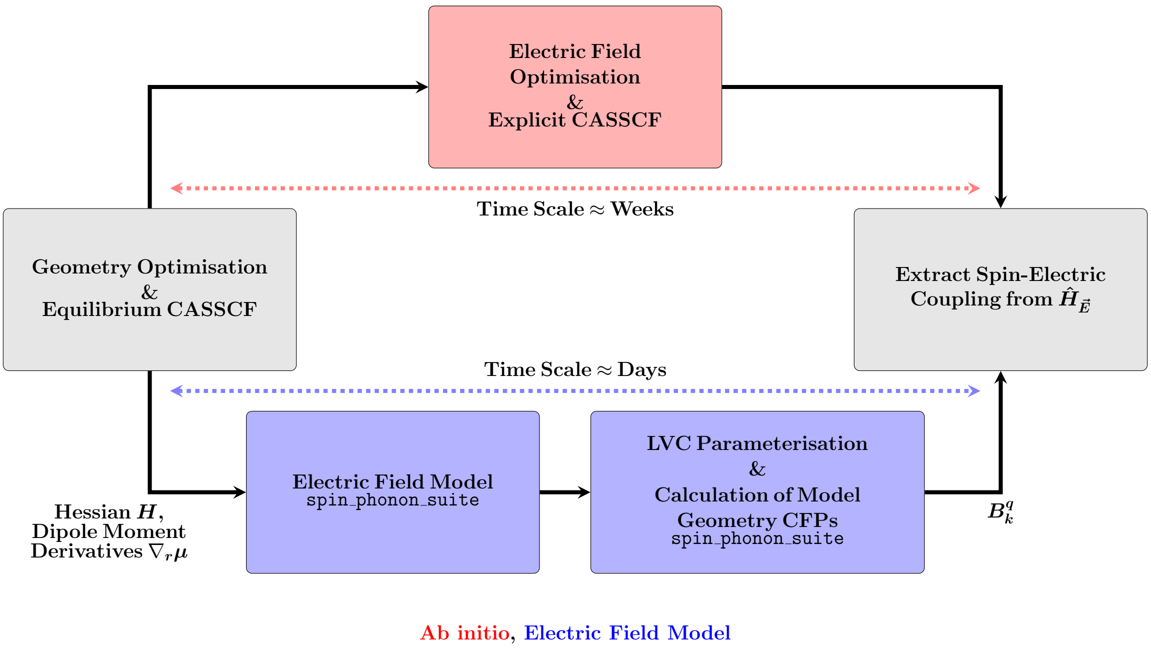

Figure S5: Flow chart representation of the work-flow from a molecules equilibrium geometry to calculating the spin-electric coupling and the inherent advantages in computational cost using our electric field model and LVC methodology in comparison to a full *ab initio* method.

S2 Perturbation Theory Derivation

To rationalise the electric and magnetic field orientation dependence observed we used perturbation theory to approximate the dependencies of the coupling elements in the electric field Hamiltonian, $\boldsymbol{H}_{\vec{E}}$. The crystal field Hamiltonian is comprised of degenerate Kramers doublet states which are even under time-reversal symmetry. Hence, structural perturbations alone will not allow for intra-Kramers doublet mixing. Therefore, the degeneracy of the doublet states must be lifted by a small perturbation in the form of a Zeeman term to allow intra-kramers doublet mixing. The *ab initio* crystal field parameters were projected into a model Hamiltonian and diagonalised into its eigenstate basis, H^0. Where the eigenvalues are doubly degenerate such that $E_1 = E_{\bar{1}} < E_2 = E_{\bar{2}} < \cdots < E_n = E_{\bar{n}}$.

Degenerate perturbation theory was used to express the ground doublet $|1\rangle$, $|\bar{1}\rangle$ as the states $|1_{\pm}\rangle$ such that there is an angle, θ, between the basis vectors, they remain orthonormal is this basis, and they remain a time-reversal conjugate doublet. The transformed basis vectors are expressed in equations (S20) and (S21).

$$|1_{+}\rangle = \cos\frac{\theta}{2}|1\rangle + e^{i\phi}\sin\frac{\theta}{2}|\bar{1}\rangle \tag{S20}$$

$$|1_{-}\rangle = -\sin\frac{\theta}{2}|1\rangle + e^{i\phi}\cos\frac{\theta}{2}|\bar{1}\rangle \tag{S21}$$

such that energy of states $|1_{\pm}\rangle$ can be expressed as $E_{1_{\pm}} = E_{1,\bar{1}} \pm \frac{\Delta E}{2}$ where ΔE is the splitting generate from the small Zeeman perturbation to the basis states as given in equation S22.

$$\Delta E = 2\sqrt{\langle 1|\boldsymbol{H}_{\text{Zee}}|1\rangle^2 + |\langle 1|\boldsymbol{H}_{\text{Zee}}|\bar{1}\rangle|^2} \tag{S22}$$

Non-degenerate perturbation theory was subsequently used to derive the corrections the states and energy from a small Zeeman term added to the total Hamiltonian. Such that in this basis the Schrodinger equation is expressed by equation (S23). Where state $|n\rangle$ is an eigenstate of the Hamiltonian.

$$(\boldsymbol{H}^0 + \boldsymbol{H}_{\text{Zee}})|n\rangle = E_n|n\rangle \tag{S23}$$

To obtain the first order correction to the energy, equation (S23) is acted on by the bra, $\langle n_0|$, which is the zeroth order correction the state $|n\rangle$. Acting with bra, $\langle n_0|$, separating the terms of the total Hamiltonian, and expanding $|n\rangle$ yields.

$$\langle n_0|\boldsymbol{H}^0|n_0\rangle + \langle n_0|\boldsymbol{H}^0|n_1\rangle + \langle n_0|\boldsymbol{H}_{\text{Zee}}|n_0\rangle + \langle n_0|\boldsymbol{H}_{\text{Zee}}|n_1\rangle + \cdots = \langle n_0|E_n|n_0\rangle + \langle n_0|E_n|n_1\rangle + \ldots \tag{S24}$$

Given that we are only going to compute the first order corrections for this analysis the terms of the equations of higher order corrections are neglected. Evaluating the expression

gives.

$$E_n^{(0)} + \langle n_0|\boldsymbol{H}_{\text{Zee}}|n_0\rangle = E_n^{(0)} + E_n^{(1)} \tag{S25}$$

$$E_n^{(1)} = \langle n_0|\boldsymbol{H}_{\text{Zee}}|n_0\rangle + \mathcal{O}(\boldsymbol{H}_{\text{Zee}}^2) + \dots \tag{S26}$$

As expected the correction to the energy of a state is approximately the expectation value of the Zeeman Hamiltonian in that state. To compute the first order correction to the states, equation (S23) is acted on by the bra, $\langle m_0|$. Where $\langle m_0|$ is the zeroth order correction of an excited state such that $m \neq 1, \bar{1}$. Again, the terms of the Hamiltonian are separated and expanded giving.

$$\langle m_0|\boldsymbol{H}^0|n_0\rangle+\langle m_0|\boldsymbol{H}^0|n_1\rangle+\langle m_0|\boldsymbol{H}_{\text{Zee}}|n_0\rangle+\langle m_0|\boldsymbol{H}_{\text{Zee}}|n_1\rangle+\dots = \langle m_0|E_n|n_0\rangle+\langle m_0|E_n|n_1\rangle+\dots \tag{S27}$$

Given that $\boldsymbol{H}^0$ is in its eigenstate basis off diagonal matrix elements are zero. Therefore, the expression above can be simplified and then rearranged to give an expression for the correction to the state.

$$\langle m_0|n_1\rangle\, E_m^{(0)} + \langle m_0|\boldsymbol{H}_{\text{Zee}}|n_0\rangle = \langle m_0|n_1\rangle\, E_n^{(0)} \tag{S28}$$

$$\langle m_0|n_1\rangle = \frac{\langle m_0|\boldsymbol{H}_{\text{Zee}}|n_0\rangle}{E_n^{(0)} - E_m^{(0)}} \tag{S29}$$

Using the the fact: $1 = \sum_{m\neq 1,\bar{1}} |m_0\rangle\,\langle m_0|$, The first order correction to the state can be expressed by equation (S30)

$$|n_1\rangle = \sum_{m_0\neq 1,\bar{1}} \frac{\langle m_0|\boldsymbol{H}_{\text{Zee}}|n_0\rangle}{E_{n_0} - E_{m_0}}\,|m_0\rangle \tag{S30}$$

Using the first order correction to the state and energy, The states $|1_\pm\rangle$ can be expressed in a new basis $|1'_\pm\rangle$ such that the states and energies are corrected for the perturbation as given in equation S32.

$$E_{1'_\pm} = E_{1_\pm} + \langle 1_\pm|\boldsymbol{H}_{\text{Zee}}|1_\pm\rangle \tag{S31}$$

$$|1'_{\pm}\rangle = |1_{\pm}\rangle + \sum_{m\neq 1,\bar{1}} \frac{\langle m|\boldsymbol{H}_{\mathrm{Zee}}|1_{\pm}\rangle}{E_1 - E_m} |m\rangle \tag{S32}$$

The correction to the state is dependent on the mixing of the ground doublet states, $|1_{\pm}\rangle$ with excited states divided by the difference in energy of those states. To compute the relative orientation dependence of the magnetic and electric fields, we project the total perturbed Hamiltonian, H' onto the corrected ground doublet derived using perturbation theory. For simpler notation, we define operator, $\boldsymbol{Q}$, that has the form $\boldsymbol{Q} = \sum_{m\neq 1,\bar{1}} \frac{|m\rangle\langle m|}{E_m - E_n}$. This allows us to write equation (S32) as equation (S34)

$$|1'_{\pm}\rangle = |1_{\pm}\rangle - \boldsymbol{Q}\boldsymbol{H}_{\mathrm{Zee}}\,|1_{\pm}\rangle \tag{S33}$$

$$|1'_{\pm}\rangle = (\boldsymbol{I} - \boldsymbol{Q}\boldsymbol{H}_{\mathrm{Zee}})\,|1_{\pm}\rangle \tag{S34}$$

The projection operator, $\boldsymbol{P}$, projects the total perturbed Hamiltonian, $\boldsymbol{H}_{\mathrm{dist}}$ onto the states $|1'_{\pm}\rangle$ and is given by equation (S35).

$$\boldsymbol{P} = |1'_{+}\rangle\,\langle 1'_{+}| + |1'_{-}\rangle\,\langle 1'_{-}| \tag{S35}$$

Projecting and evaluating the known terms gives equation (S36).

$$\boldsymbol{P}\boldsymbol{H}_{\mathrm{dist}}\boldsymbol{P} = \underbrace{\begin{bmatrix} E_1 - \frac{\Delta E}{2} & 0 \\ 0 & E_{\bar{1}} + \frac{\Delta E}{2} \end{bmatrix}}_{\boldsymbol{P}(\boldsymbol{H}_{\mathrm{CF}}+\boldsymbol{H}_{\mathrm{Zee}})\boldsymbol{P}} + \underbrace{\begin{bmatrix} \langle 1'_{+}|\boldsymbol{H}_{\vec{E}}|1'_{+}\rangle & \langle 1'_{+}|\boldsymbol{H}_{\vec{E}}|1'_{-}\rangle \\ \langle 1'_{-}|\boldsymbol{H}_{\vec{E}}|1'_{+}\rangle & \langle 1'_{-}|\boldsymbol{H}_{\vec{E}}|1'_{-}\rangle \end{bmatrix}}_{\boldsymbol{P}\left(\boldsymbol{H}_{\vec{E}}\right)\boldsymbol{P}} \tag{S36}$$

The off-diagonal spin-electric coupling terms are only found in the projection of the electric field Hamiltonian onto the ground doublet. Therefore, the decomposition of the spin-electric coupling can be found by expressing the off-diagonal matrix elements in the unperturbed basis using the equation (S34).

$$\langle 1'_{\pm}|\boldsymbol{H}_{\vec{E}}|1'_{\mp}\rangle = \langle 1'_{\pm}|\boldsymbol{H}_{\mathrm{dist}}|1'_{\mp}\rangle = \langle 1_{\pm}|\,(\boldsymbol{I} - \boldsymbol{H}_{\mathrm{Zee}}\boldsymbol{Q})\,\boldsymbol{H}_{\vec{E}}\,(\boldsymbol{I} - \boldsymbol{Q}\boldsymbol{H}_{\mathrm{Zee}})\,|1_{\mp}\rangle \tag{S37}$$

Evaluating inside the braket gives (noting that on the left hand side the basis states are

primed and they are non-primed on the right hand side)

$$\langle 1'_{\pm}|\boldsymbol{H}_{\vec{E}}|1'_{\mp}\rangle = \langle 1'_{\pm}|\boldsymbol{H}_{\text{dist}}|1'_{\mp}\rangle = \underbrace{\langle 1_{\pm}|H_{\vec{E}}|1_{\mp}\rangle}_{0} - \underbrace{\langle 1_{\pm}|\boldsymbol{H}_{\text{Zee}}\boldsymbol{Q}\boldsymbol{H}_{\vec{E}} + \boldsymbol{H}_{\vec{E}}\boldsymbol{Q}\boldsymbol{H}_{\text{Zee}}|1_{\mp}\rangle}_{A} + \mathcal{O}(\boldsymbol{H}^2_{\text{Zee}}) \tag{S38}$$

$$\langle 1'_{\pm}|\boldsymbol{H}_{\vec{E}}|1'_{\mp}\rangle = \langle 1'_{\pm}|\boldsymbol{H}_{\text{dist}}|1'_{\mp}\rangle \approx -\langle 1_{\pm}|\boldsymbol{H}_{\text{Zee}}\boldsymbol{Q}\boldsymbol{H}_{\vec{E}} + \boldsymbol{H}_{\vec{E}}\boldsymbol{Q}\boldsymbol{H}_{\text{Zee}}|1_{\mp}\rangle \tag{S39}$$

Where A can be expanded using equation (S34) to reform the full expression for the off-diagonal matrix elements. $\mathcal{O}(\boldsymbol{H}^2_{\text{Zee}})$ is omitted as its second order in the Zeeman term.

$$\langle 1'_{\pm}|\boldsymbol{H}_{\vec{E}}|1'_{\mp}\rangle = \langle 1'_{\pm}|\boldsymbol{H}_{\text{dist}}|1'_{\mp}\rangle \approx -\sum_{m\neq 1,\bar{1}} \frac{\langle 1_{\pm}|\boldsymbol{H}_{\text{Zee}}|m\rangle\langle m|\boldsymbol{H}_{\vec{E}}|1_{\mp}\rangle}{E_m - E_1} + \frac{\overbrace{\langle 1_{\pm}|\boldsymbol{H}_{\vec{E}}|m\rangle}^{-\langle \bar{m}|(-\boldsymbol{H}_{\vec{E}})|1_{\mp}\rangle}\overbrace{\langle m|\boldsymbol{H}_{\text{Zee}}|1_{\mp}\rangle}^{\langle 1_{\pm}|\boldsymbol{H}_{\text{Zee}}|m\rangle}}{E_m - E_1} \tag{S40}$$

Combining the summation gives the perturbative expression seen in the main text.

$$\langle 1'_{\pm}|\boldsymbol{H}_{\vec{E}}|1'_{\mp}\rangle = \langle 1'_{\pm}|\boldsymbol{H}_{\text{dist}}|1'_{\mp}\rangle \approx -2\sum_{m\neq 1,\bar{1}} \frac{\langle 1_{\pm}|\boldsymbol{H}_{\vec{E}}|m\rangle\langle m|\boldsymbol{H}_{\text{Zee}}|1_{\mp}\rangle}{E_m - E_1} \tag{S41}$$

Magnetic and Electric Field Orientation Dependence of the Spin-Electric Coupling

To understand the magnetic field orientation dependence of the spin-electric coupling the magnetic anisotropy (**Figure S2**) of excited Kramers doublets need to be considered as perturbation theory shows that the spin-electric coupling between the ground Kramers doublet is dependent on the mixing of the ground Kramers doublets with excited Kramers doublets. The magnetic anisotropy of excited Kramers doublets becomes increasingly easy-axis along x for as the doublet energy increases (**Table S2**). The Zeeman Hamiltonian's for each magnetic field orientation (**Figure S7b**) shows that when the magnetic field is orientated along the main molecular axis x, there are intra-Kramers doublet mixing which break the degeneracy of the Kramers doublets and near-zero inter-Kramers doublet mixing. For excited Kramers doublets that have easy-axis anisotropy we can chose that the states $|m\rangle$ and $|\bar{m}\rangle$ each correspond to $|\boldsymbol{J}_x; m_J\rangle$ states such that they are

eigenstates of $\boldsymbol{J}_x$ which have the freedom to rotate within Kramers doublet forming linear combinations and hence there is only off diagonal matrix elements within each Kramers doublets. On the other hand, when the magnetic field is orientated in the easy-plane (y, z) we observe only inter-Kramers doublet mixing as the excited states are not eigenstates of $\boldsymbol{J}_y$ or $\boldsymbol{J}_z$ and we see off diagonal matrix elements between the the ground Kramers doublet and Kramers doublets with $|\boldsymbol{J}_x; m_J\rangle$ representations of $|\boldsymbol{J}_x; m_J \pm 1\rangle$. This coincides with the magnetic field orientation dependence seen in *ab initio*, as the perturbation theory shows that the spin-electric coupling is dependent on the inter-Kramers doublet mixing. As there is only intra-Kramers doublet mixing when the magnetic field is orientated along x this explains the smallest spin-electric coupling.

Perturbation theory can also be used to rationalise the electric field orientation dependence. The electric field Hamiltonian in the eigenbasis of the equilibrium geometry crystal field Hamiltonian (**Figure S7a**) for each electric field orientation all show zero intra-Kramers doublet mixing as the doublets in this basis are degenerate and are related by time-reversal conjugate symmetry. In the case where the electric field is orientated along (pseudo)-high-symmetry axis (y) where the distortion is symmetry preserving the electric field Hamiltonian is near-diagonal such that this distortion purely shifts the energies of the crystal field eigenstates. However, when the electric field is orientated perpendicular to the (pseudo)-high-symmetry axis where the distortion are always symmetry breaking there are inter-Kramers doublet mixing elements which support the *ab initio* findings the electric field orientated perpendicular to the high-symmetry axis generate larger spin-electric coupling.

The comparison of spin-electric couplings derived from perturbation theory with the spin-electric couplings from *ab initio* and the electric field model are shown in figure S6. Perturbation theory correctly predicts the magnetic field orientation dependence for E_x and E_z when comparing to *ab initio* with B_y and B_z having very good agreement. However perturbation theory under-predicts the spin-electric coupling for B_x by approximately

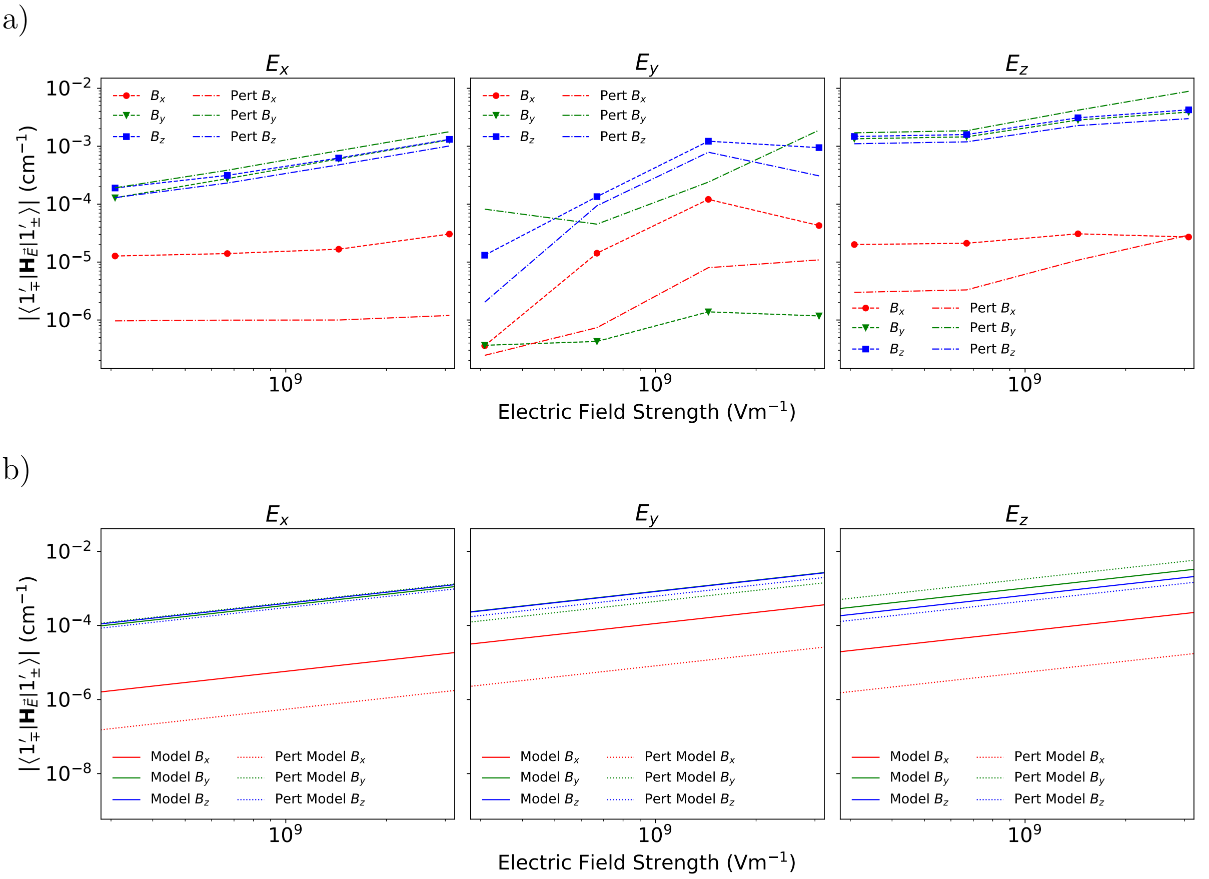

Figure S6: The spin-electric coupling as a function of field strength comparing pure *ab initio* (a), the electric field model and LVC (b) with the spin-electric couplings derived from perturbation theory using a magentic field strength of 32 mT.

one order of magnitude. Perturbation theory doesn't well reproduce the spin-electric coupling for E_y where the electric field Hamiltonian is diagonal in the eigenstate basis of the equilibrium geometry crystal field Hamiltonian. We observe better agreement when comparing perturbation theory to the electric field model, this can be attributed to the electric field model not preserving the pseudo-C_2 symmetry when the electric field is orientated along the high-symmetry axis. We note that perturbation theory only holds for small perturbations in the Zeeman Hamiltonian. Therefore, when comparing the spin-electric couplings we apply magnetic fields with magnitudes one order of magnitude smaller (32 mT) than we use for calculating the spin-electric couplings in the main text.

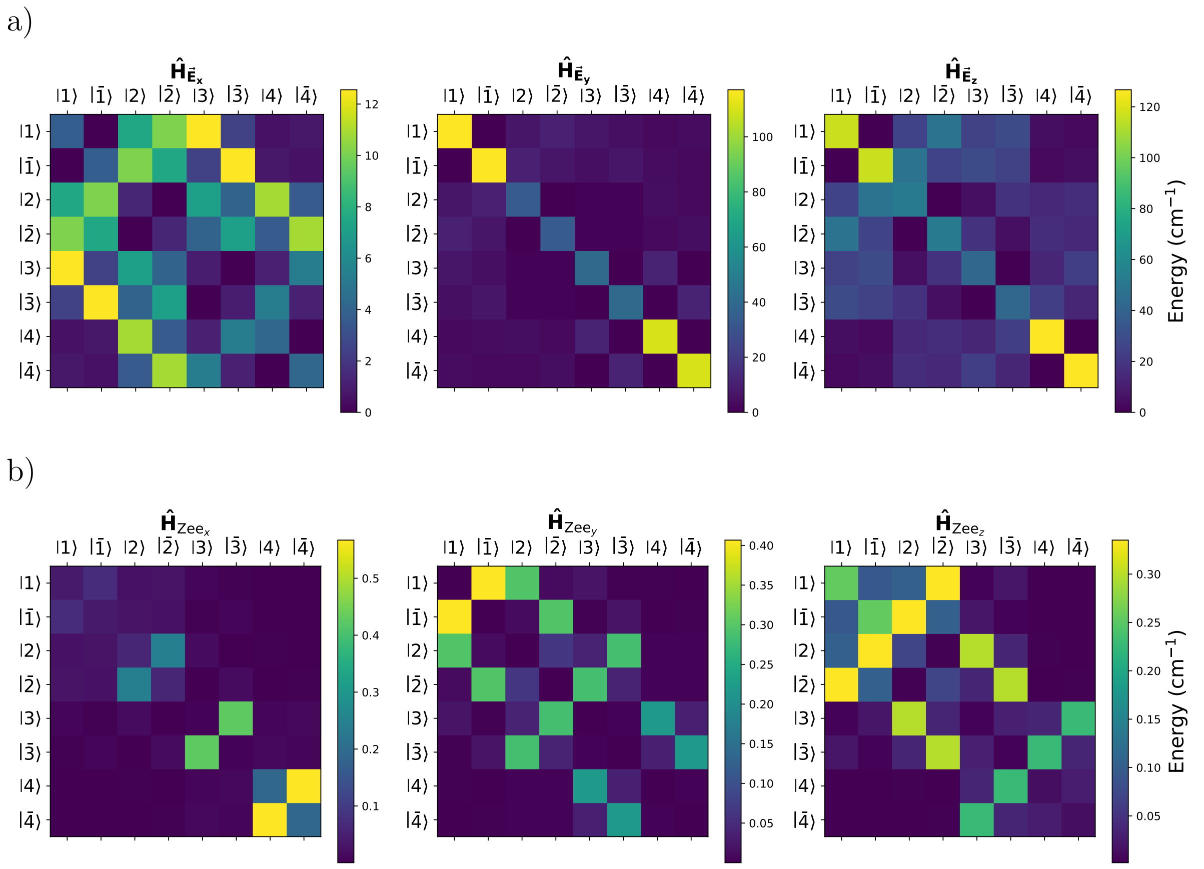

Figure S7: Heat-map representations of the electric field (a) and Zeeman (b) Hamiltonian's in the eigenstate basis of the equilibrium geometry crystal field Hamiltonian. Such that the matrix elements $\langle m|\boldsymbol{H}_{\mathrm{Zee}}|1_{\mp}\rangle$ and $\langle 1|\boldsymbol{H}_{\vec{E}}|m\rangle$ from perturbation theory are the off diagonal matrix elements of the electric field and Zeeman Hamiltonian's shown.

S3 Spin dynamics driven with resonant frequency electric fields.

The proposed experiment would manipulate the molecular spin using a resonant frequency electric field pulse. Where the resonant frequency (ω) is calculated from the splitting of the ground doublet due to the weak static magnetic field.

$$\omega = \frac{\left|\langle \boldsymbol{H}_{\mathrm{eq}}\rangle_{1'_-} - \langle \boldsymbol{H}_{\mathrm{eq}}\rangle_{1'_+}\right|}{\hbar} \tag{S42}$$

where ω is the resonant frequency that drives spin population between states $|1'_-\rangle$ and $|1'_+\rangle$. To model these dynamics, we use evolve a spin population density matrix using the Liouville-Von Neumann equation which is defined as the commutator between the time

dependent Hamiltonian and the spin density matrix.

$$\frac{\partial \boldsymbol{\rho}(t)}{\partial t} = -\frac{i}{\hbar}\left[\boldsymbol{H}(t), \boldsymbol{\rho}(t)\right] \tag{S43}$$

$$= -\frac{i}{\hbar}\left(\boldsymbol{H}(t)\cdot\boldsymbol{\rho}(t) - \boldsymbol{\rho}(t)\cdot\boldsymbol{H}(t)\right) \tag{S44}$$

Where $\boldsymbol{H}(t)$ is the total time dependent Hamiltonian of the system at time t, which includes the equilibrium Hamiltonian ($\boldsymbol{H}_{\text{eq}}$) and the oscillating electric field Hamiltonian ($\boldsymbol{H}_{\vec{E}}$) as shown in equation (S45).

$$\boldsymbol{H}(t) = \boldsymbol{H}_{\text{eq}} + \boldsymbol{H}_{\vec{E}}(t)\sin(\omega t) \tag{S45}$$

We write equation (S43) such that the density matrix $\boldsymbol{\rho}(t)$ is a column vector noting that $\boldsymbol{A\rho B} = \left(\boldsymbol{B}^T \otimes \boldsymbol{A}\right)\vec{\rho}$. Hence the dynamics of the system can be solved using an ordinary differential equation (ODE) solver rather than using explicit numerical integration at each time step. The modified Liouville-Von Neuamn equation is given by:

$$\frac{\partial \vec{\rho}(t)}{\partial t} = \frac{-i}{\hbar}(\boldsymbol{I}\otimes\boldsymbol{H}(t) - \boldsymbol{H}(t)^T\otimes\boldsymbol{I})\vec{\rho}(t) \tag{S46}$$

$$= \frac{-i}{\hbar}\left(\boldsymbol{I}\otimes(\boldsymbol{H}_{\text{eq}} + \boldsymbol{H}_{\vec{E}}\sin(\omega t)) - \left(\boldsymbol{H}_{\text{eq}}^T + \boldsymbol{H}_{\vec{E}}^T\sin(\omega t)\right)\otimes\boldsymbol{I}\right)\vec{\rho}(t) \tag{S47}$$

The transition probability between states $|i\rangle$ and $|j\rangle$ ($|c_{ij}(t)|^2$) shown in figure S8c as a function of pulse duration and driving frequency detuning was calculated using equation (S48). Where Ω_{ij} is the Rabi frequency of the transition between $|i\rangle$ and $|j\rangle$, and δ is the detuning of the drive frequency.

$$|c_{ij}(t)|^2 = \frac{\Omega_{ij}^2\sin^2\left(\frac{\sqrt{\Omega_{ij}^2+\delta^2}t}{2}\right)}{\Omega_{ij}^2+\delta^2} \tag{S48}$$

Where the Rabi frequency Ω_{ij} of the transition between states $|i\rangle$ and $|j\rangle$ is calculated using equation (S49).

$$\Omega_{ij} = \frac{|\langle i|\boldsymbol{H}_{\vec{E}}|j\rangle|}{\hbar} \tag{S49}$$

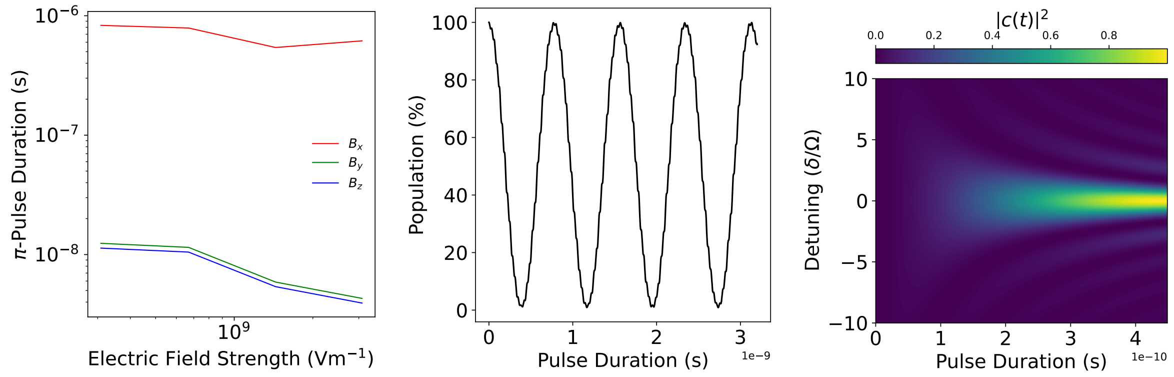

Figure S8: a) The pulse duration required to perform a π-pulse as a function of field strength for E_z and all magnetic field orientations using the spin-electric couplings calculated *ab initio*. b) The Rabi oscillation of state $|1'_{-}\rangle$. The simulations used an applied electric field magnitude of $3.08 \times 10^{9}\,\mathrm{V\,m^{-1}}$ and a magnetic field magnitude of 320 mT both orientated along the molecular z axis and a driving frequency of 1×10^{11} Hz. c) The transition probability of the transition from state $|1'_{-}\rangle$ to state $|1'_{+}\rangle$ as a function of time and detuning of the resonant drive frequency.

We note our dynamics do not fully reproduce experimental conditions for a number of reasons. Firstly, in our π-pulse simulation our dynamics are unitary and neglect spin decoherence due to spin-phonon coupling. Secondly, we make a number of assumptions in our oscillating electric field Hamiltonian term. Optimisation of the molecular geometry or using our analytical model produce the equilibrium geometry in the applied electric field. However, these optimisations do not account for the time scale of molecular distortion. Hence, when the electric field Hamiltonian oscillates in our simulation we make the assumption that in the period of ω our geometry can distort from the equilibrium zero-field geometry to the maximum distorted geometry at that specific electric field strength. Finally, our simulation makes the assumption of a perfectly uniform pulse, i.e. we do not include any shaping to our pulse.

S4 Pseudo C_2 Irrep Symmetry Decomposition

Distortions can not only be written as a linear combination of normal mode displacements but can be written as a linear combination of any arbitrary orthonormal basis. To provide further insight into the type of distortion that has the largest influence on spin-electric coupling. The distortion due to an applied electric field can be decomposed into

transformed into a symmetry adapted coordinate basis. Where a set of displacement vectors are constructed such that (pseudo)-equivalent atoms related by (pseudo)-symmetry are simultaneously displaced in in-phase and out-of-phase displacements, i.e. each displacement is categorised by its definite pseudo-symmetry irreducible representation of the point group. These explicit displacements are orthonormalised using the Gram-Schmidt procedure, which takes the set of linear independent displacement vectors that span the $3N$ nuclear degrees of freedom and generates a set of orthonormal basis vectors of the same $3N$ dimensions. This forms the new symmetry adapted coordinate basis, $\boldsymbol{Z}$. Where the basis vectors are categorised into their corresponding pseudo-symmetry irrep, that consists of symmetry breaking (B symmetry) and symmetry preserving (A symmetry) orthogonal coordinate basis vectors. Furthermore, energy invariant rigid-body rotations and translations are removed as these displacements do not contribute to geometric distortions. The distorted structure due to an electric field, $\boldsymbol{r_E}$, can be written as a liner combination of both A and B symmetry distortions.

$$\boldsymbol{r_E} = \boldsymbol{r}_{\text{eq}} + \sum_{i}^{n_a} a_i \boldsymbol{A_i} + \sum_{j}^{n_b} b_j \boldsymbol{B_j} \quad = \quad \boldsymbol{r}_0 + \sum_{i}^{N} z_i \boldsymbol{Z_i} \tag{S50}$$

The symmetry adapted coordinate basis $\boldsymbol{Z}$ which has the matrix form.

$$\vec{z} = \begin{bmatrix} \vec{a} \\ \vec{b} \end{bmatrix}^T \qquad \boldsymbol{Z} = \begin{bmatrix} \boldsymbol{A} \\ \boldsymbol{B} \end{bmatrix} \tag{S51}$$

Using the new orthonormal $\boldsymbol{Z}$ basis the magnitude of each displacement vector, $\vec{z}$, can be projected.

$$\left(\vec{r}_{\vec{E}} - \vec{r}_{\text{eq}}\right) \boldsymbol{Z_j} = \sum_{i}^{N} z_i \underbrace{\boldsymbol{Z_i} \cdot \boldsymbol{Z_j}}_{\delta_{ij}} \tag{S52}$$

$$\boldsymbol{Z}^T \left(\vec{r}_{\vec{E}} - \vec{r}_{\text{eq}}\right) = \boldsymbol{Z}^T \boldsymbol{Z} \cdot \vec{z} \tag{S53}$$

As the matrix $\boldsymbol{Z}$ is an orthogonal basis $\boldsymbol{Z}\boldsymbol{Z^T} = \boldsymbol{I}$ such that we recover a vector of displacement magnitudes $\boldsymbol{z}$ which are redistributed into $\vec{a}, \vec{b}$ using equation (S51). A set

of $3N-6$ structures are then produced with a single distortion displaced.

$$r_{A,i} = \vec{r}_{\text{eq}} + a_i \boldsymbol{A}_i, \qquad \vec{r}_{B,j} = \vec{r}_{\text{eq}} + b_i \boldsymbol{B}_j \tag{S54}$$

The crystal field parameters were extracted using the Linear Vibronic Coupling model and projected onto a crystal field Hamiltonian. Rotating into the equilibrium geometries eigenbasis allows for the computation of the contribution each distortion has to the total spin-electric coupling and total contribution from symmetry preserving A distortions and symmetry breaking B distortions.

$$\langle 1_\pm | \boldsymbol{H'} | 1_\mp \rangle \approx \sum_i \langle 1_\pm | \boldsymbol{H_{A_i}} | 1_\mp \rangle + \sum_j \langle 1_\pm | \boldsymbol{H_{B_j}} | 1_\mp \rangle \tag{S55}$$

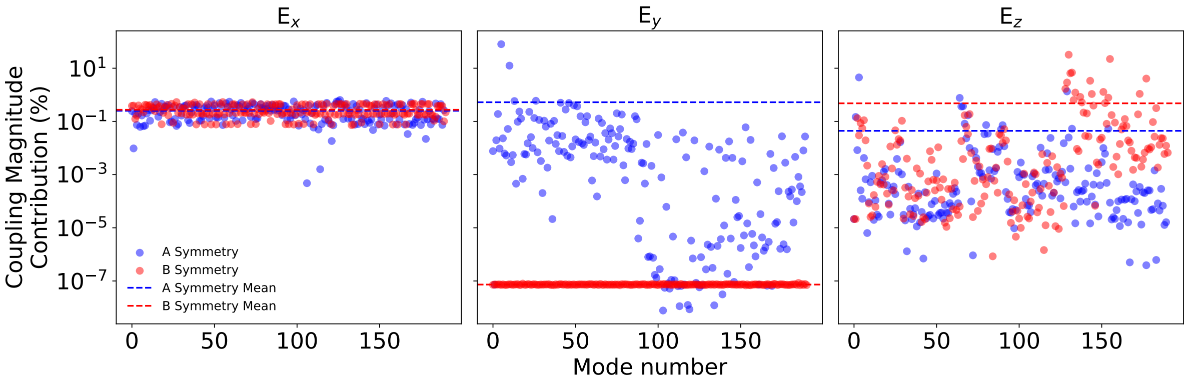

Figure S9: The contribution to the total spin-electric coupling magnitude due to an applied electric field along each Cartesian orientation (3×10^9 V m^{-1}) between the ground Kramers doublet split by the Zeeman effect due to a magnetic field aligned along Cartesian x (320 mT) for each 'A' symmetry mode and each 'B' symmetry mode in the symmetry adapted coordinate basis for the pseudo-C_2 point group.

S5 Electronic Structure

Calculated principal g values for $Tm(N^{\dagger\dagger})_2$ optimised in the gas phase shows good agreement with experimental values, (1.15, 3.64, 5.46), only differing in the choice of notation for G_z which conventionally is assigned to the largest g value. However, we have chosen to report our g values in accordance to the arbitrary Cartesian coordinate directions to aid analysis. Calculations correctly predict the easy-plane magnetic anisotropy. The spin

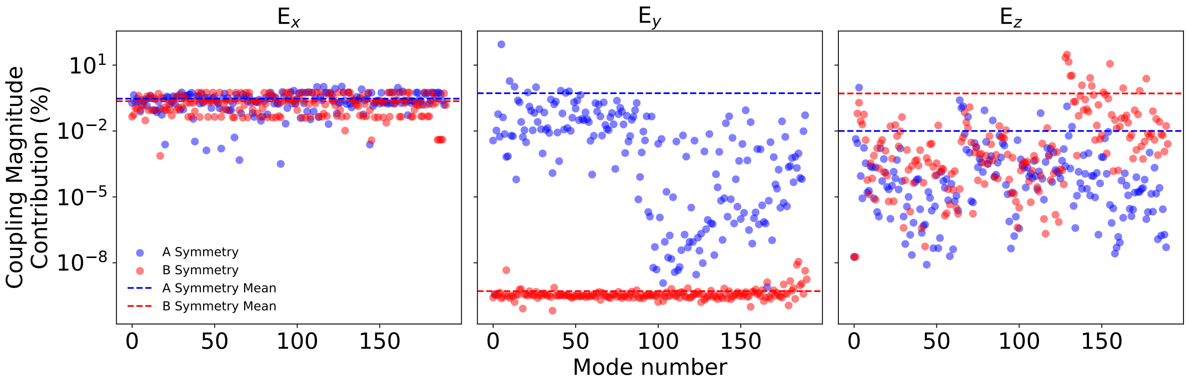

Figure S10: The contribution to the total spin-electric coupling magnitude due to an applied electric field along each Cartesian orientation (3×10^9 V m^{-1}) between the ground Kramers doublet split by the Zeeman effect due to a magnetic field aligned along Cartesian y (320 mT) for each 'A' symmetry mode and each 'B' symmetry mode in the symmetry adapted coordinate basis for the pseudo-C$_2$ point group.

Table S2: Crystal field states of the equilibrium geometry of Tm(N††)$_2$. Where the principal g-values are given in the molecular reference to aid in the discussion of the magnetic field orientation dependence of the spin-electric coupling in the main text.

Molcas Energy (cm^{-1})	Crystal Field Energy (cm^{-1})	Principal G values Gx	Gy	Gz	Crystal field wavefunction
0.00	0.00	1.15	5.44	3.67	97.61 % $\lvert\pm1/2\rangle$
549.54	549.54	3.45	0.84	0.92	96.67 % $\lvert\pm3/2\rangle$
1299.20	1299.20	5.76	0.05	0.06	98.30 % $\lvert\pm5/2\rangle$
2270.42	2270.42	8.00	0.05	0.05	99.53 % $\lvert\pm7/2\rangle$

orbit states are well represented by as projections of the total angular momentum, $\langle \boldsymbol{J}_x \rangle$, and show a bistable $m_j = \pm 1/2$ ground state.

S6 *Ab Initio* Spin-electric Couplings

Table S3: Spin electric coupling values calculated from *ab initio* for each electric and magnetic file orientation, for each electric field strength, and for a magnetic field strength of 320 mT

$\vec{E}$ orientation	$\|\vec{E}\|$ (V m^{-1})	$\langle 1_\pm \| \boldsymbol{H}_{\vec{E}} \| 1_\mp \rangle$ B_x (cm^{-1})	$\langle 1_\pm \| \boldsymbol{H}_{\vec{E}} \| 1_\mp \rangle$ B_y (cm^{-1})	$\langle 1_\pm \| \boldsymbol{H}_{\vec{E}} \| 1_\mp \rangle$ B_z (cm^{-1})
x	3.085×10^8	1.273×10^{-4}	1.286×10^{-3}	1.901×10^{-3}
x	6.685×10^8	1.402×10^{-4}	2.756×10^{-3}	3.135×10^{-3}
x	1.440×10^9	1.667×10^{-4}	6.060×10^{-3}	6.251×10^{-3}
x	3.085×10^9	3.046×10^{-4}	1.290×10^{-2}	1.313×10^{-2}
y	3.085×10^8	3.592×10^{-6}	3.660×10^{-6}	1.314×10^{-4}
y	6.685×10^8	1.422×10^{-4}	4.266×10^{-6}	1.347×10^{-3}
y	1.440×10^9	1.209×10^{-3}	1.382×10^{-5}	1.219×10^{-2}
y	3.085×10^9	4.270×10^{-4}	1.176×10^{-5}	9.451×10^{-3}
z	3.085×10^8	2.008×10^{-4}	1.343×10^{-2}	1.472×10^{-2}
z	6.685×10^8	2.113×10^{-4}	1.451×10^{-2}	1.590×10^{-2}
z	1.440×10^9	3.076×10^{-4}	2.834×10^{-2}	3.096×10^{-2}
z	3.085×10^9	2.707×10^{-4}	3.884×10^{-2}	4.248×10^{-2}
